# Environmental Contaminants and Osteoporosis-Related Outcomes: A Systematic Review and Meta-Analysis

**DOI:** 10.3390/toxics14090790

**Published:** 2026-09-07

**Authors:** Francesco Leonforte, Vito Nicosia, Gianluca Testa, Marco Sapienza, Fabrizio Mattu, Alessia Caldaci, Tommaso Filippini, Vito Pavone, Antonio Mistretta

**Affiliations:** 1 Structural Department, Health Management, University Hospital Polyclinic “G. Rodolico—San Marco”, 95123 Catania, Italy; leonfortefrancesco1@gmail.com; 2 Department of Medical, Surgical Sciences and Advanced Technologies “G.F. Ingrassia”, University of Catania, 95123 Catania, Italy; anmist@unict.it; 3 Department of General Surgery and Medical Surgical Specialties, University Hospital Polyclinic “G. Rodolico—San Marco”, 95123 Catania, Italy; marcosapienza09@yahoo.it (M.S.); alessia.c.92@hotmail.it (A.C.); vpavone@unict.it (V.P.); 4 Department of Medical Science and Public Health, University of Cagliari, 09042 Monserrato, Italy; fabrimattu@gmail.com; 5 Environmental, Genetic and Nutritional Epidemiology Research Center (CREAGEN), Department of Biomedical, Metabolic and Neural Sciences, University of Modena and Reggio Emilia, 41125 Modena, Italy; tommaso.filippini@unimore.it; 6 School of Public Health, University of California Berkeley, Berkeley, CA 94074, USA; 7 Scientific Communication Service, National Institute of Public Health (Istituto Superiore di Sanità), 00161 Roma, Italy

**Keywords:** environmental contaminants, particulate matter, osteoporosis, bone mineral density, public health, meta-analysis

## Abstract

Environmental contaminants (ECs) may impair skeletal health through endocrine disruption, oxidative stress, inflammation, and altered mineral homeostasis. This systematic review and meta-analysis evaluated the associations between EC exposure and osteoporosis-related outcomes in adults. PubMed/MEDLINE, Scopus, and Web of Science were searched in April 2026 for English-language studies published from 2016 onward. Study selection followed PRISMA guidelines, while methodological quality was evaluated using the Joanna Briggs Institute (JBI) checklist and Newcastle–Ottawa Scale (NOS). Evaluated contaminant classes encompassed ambient air pollution (PM2.5, PM10, PM1, NO_2_, NO_x_, SO_2_, CO, O_3_), per- and polyfluoroalkyl substances (PFAS), heavy metals and trace elements (e.g., cadmium, lead), pesticides, polycyclic aromatic hydrocarbons (PAHs), phthalates, volatile organic compounds (VOCs), persistent organic pollutants (POPs, dioxins, PCBs), brominated flame retardants (BFRs), and water disinfection by-products. Random-effects meta-analyses using restricted maximum likelihood estimation were conducted when at least 3 studies were available. Forty-eight studies involving 4,510,868 participants were included. Air contaminants showed the most consistent associations with reduced bone mineral density (BMD) and osteoporosis, followed by selected PFAS and cadmium exposures. Evidence for pesticides, PAHs, phthalates, volatile organic compounds VOCs, POPs, flame retardants, and water disinfection by-products was less consistent. PM2.5 exposure was associated with increased osteoporosis risk (OR = 1.24, 95% CI: 1.02–1.51), and to a lesser extend also PM10 (OR = 1.17, 95% CI: 0.96–1.42). The analyses showed extreme heterogeneity and sensitivity to influential studies. ECs, particularly fine particulate matter, may contribute to skeletal deterioration, although the findings require cautious interpretation. PROSPERO ID: CRD420261460078.

## 1. Introduction

Environmental contaminants (ECs) are increasingly recognized as contributors to chronic non-communicable diseases because of their widespread distribution, persistence, and ability to interfere with biological systems even at low exposure levels [1,2]. Human exposure occurs through inhalation of polluted air, ingestion of contaminated food or drinking water, dermal contact, and occupational or residential exposure [3,4].

In this context, the translation of environmental risk perception into public health awareness is mediated by communication channels and population literacy, where media ecosystems and sociodemographic moderators strongly influence how health threats and preventative behaviors are understood and acted upon [5]. In real-life settings, individuals are usually exposed to complex mixtures of ambient air contaminants, per- and polyfluoroalkyl substances (PFAS), metals, pesticides, plasticizers, volatile organic compounds (VOCs), persistent organic pollutants (POPs), brominated flame retardants (BFRs), water disinfection by-products, and other emerging contaminants [6,7,8]. Although these exposures have been widely studied in relation to respiratory, cardiovascular, metabolic, endocrine, reproductive, and neurodevelopmental outcomes, their contribution to skeletal damage remains less clearly defined [9,10]. Bone is a dynamic tissue regulated by endocrine, inflammatory, oxidative, renal, nutritional, and mechanical factors, all of which may be affected by ECs. Fine particulate matter and traffic-related contaminants may promote systemic inflammation, oxidative stress, endothelial dysfunction, and vitamin D-related alterations [11]. PFAS may interfere with thyroid function, sex steroid signaling, lipid metabolism, and nuclear receptor-mediated pathways [12]. Metals such as cadmium and lead may accumulate in bone or disrupt mineral homeostasis, whereas pesticides, polycyclic aromatic hydrocarbons (PAHs), phthalates, VOCs, aldehydes, dioxins, PCBs, and other persistent contaminants may exert endocrine-disrupting, pro-inflammatory, mitochondrial, or epigenetic effects [13].

Growing attention has been directed toward the potential role of ECs in skeletal health [14]. However, the available evidence remains heterogeneous because contaminant classes, exposure assessment methods, study populations, and bone-related outcomes differ substantially across studies. Among the exposures examined, air pollution has received comparatively greater research attention, whereas evidence for several other ECs remains more limited and fragmented.

Therefore, this systematic review aimed to synthesize the available evidence on the relationship between ECs and osteoporosis-related outcomes, assessing the overall impact of environmental exposures on bone mineral density (BMD) and osteoporosis, and comparing the skeletal effects of different contaminant classes. When sufficiently comparable data are available, a meta-analysis will be performed to obtain pooled estimates.

## 2. Methods

### 2.1. Overview and Protocol Registration

This systematic review and meta-analysis were designed and conducted following the Preferred Reporting Items for Systematic Reviews and Meta-Analyses (PRISMA) guidelines (see Supplementary Materials Section). The protocol has been officially registered in the international PROSPERO database, under the number CRD420261460078, ensuring transparency and methodological traceability. All methodological steps were designed to guarantee reproducibility, minimize bias, and maintain a high standard of scientific rigor throughout the review process [15].

### 2.2. Eligibility Criteria

Inclusion and exclusion criteria were established a priori according to the PECO-S framework:

- **Population:** Adult population aged 18 and older of any geographical origin, with no restrictions applied to sex or ethnicity. Studies were excluded if they were experimental animal models, in vitro or in silico mechanistic evaluations, or if they were conducted only on children and pregnant women.
- **Exposure:** Human exposure to prioritized chemical ECs. Contaminant classes were selected based on known ubiquity in human living environments and biological plausibility for osteotoxicity. These classes specifically included: ambient air pollution (particulate matter and combustion gases), PFAS, toxic metals and trace elements, pesticides, PAHs, plasticizers (phthalates, bisphenols), VOCs, POPs, BFRs, micro- and nanoplastics and water disinfection by-products. Human exposure was operationally defined through objective quantification, comprising certified biomonitoring across specific biological matrices (blood, serum, or standardized urine), high-resolution spatiotemporal environmental modeling, validated dietary assessment tools, and specific genetic variants.
- **Comparator:** Populations exposed to lower levels or reference concentrations of the respective ECs.
- **Outcome:** Skeletal health outcomes explicitly related to osteoporosis, including direct surrogate markers like BMD, t-scores, z-scores, and clinical endpoints such as osteoporotic fractures.
- **Study Design:** Peer-reviewed observational and experimental studies (including prospective or retrospective cohort studies, case–control studies, and cross-sectional designs). Review articles, editorials, commentaries, case reports were excluded.

### 2.3. Information Sources and Search Strategy

A comprehensive literature search was independently performed across following electronic biomedical databases: PubMed/MEDLINE, Scopus, and Web of Science. Search was performed on 30 April 2026 and only articles published from 2016 were considered to capture the contemporary era of environmental epidemiology. The search architecture utilized a sensitive combination of Medical Subject Headings (MeSH) terms and free-text keywords tailored to each database and connected with Boolean operators. Only articles published in English language were considered.

### 2.4. Selection Process and Data Extraction

**Figure 1:**
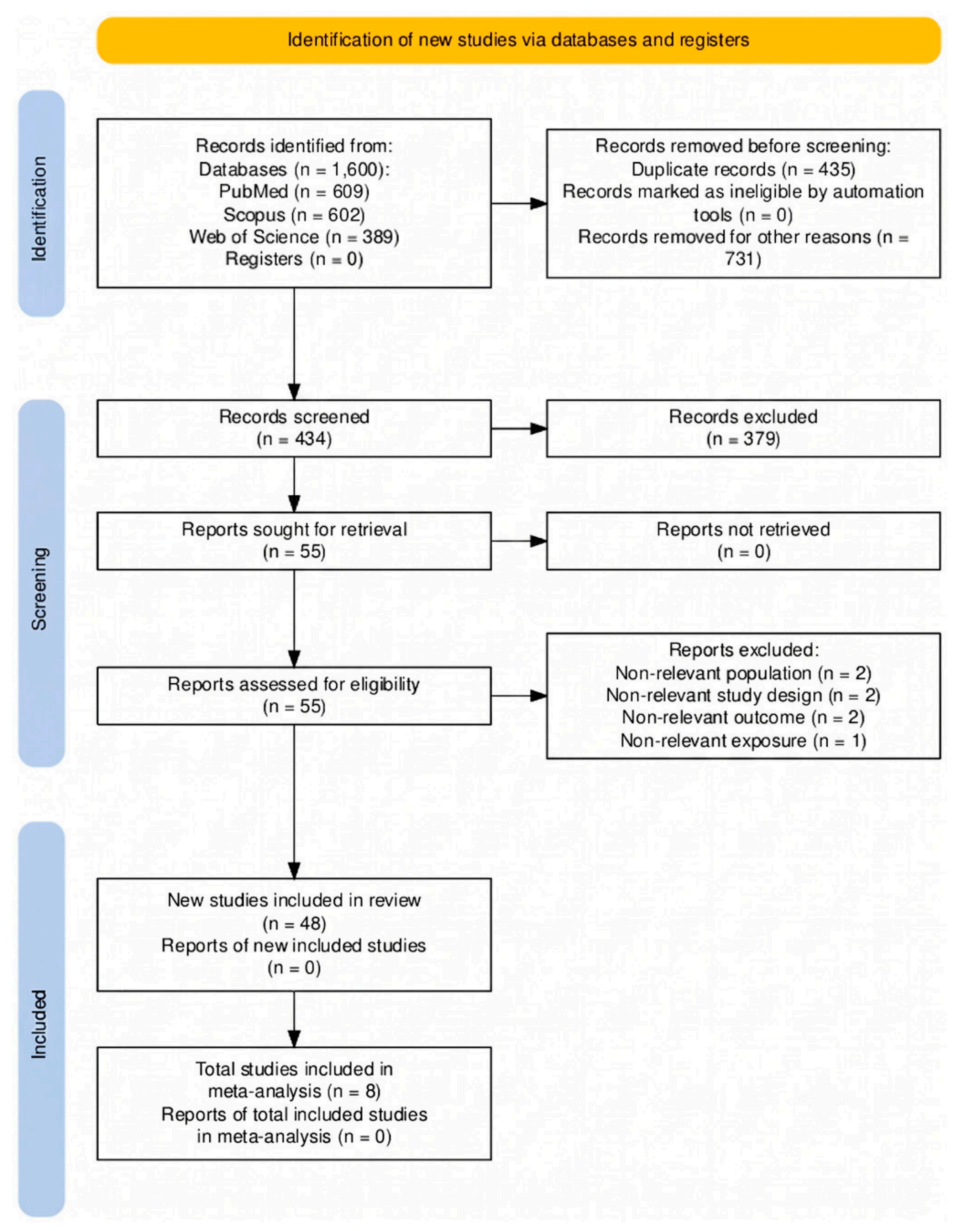
PRISMA 2020 flow diagram illustrating the literature search and study selection process. A total of 1600 records were initially identified from three electronic databases: PubMed (n = 609), Scopus (n = 602), and Web of Science (n = 389). After the removal of 435 duplicates and 731 records for other reasons, 434 records were screened. Following the exclusion of 379 records during the screening phase, 55 reports were sought for retrieval and successfully obtained. These 55 reports were assessed for eligibility, resulting in the exclusion of 6 records due to non-relevant population (n = 2), study design (n = 2), outcome (n = 2), or exposure (n = 1). Consequently, 48 new studies were included in the systematic review, and 8 of these studies were ultimately included in the meta-analysis.

The study selection process was performed using the Rayyan^®^ platform (Rayyan Systems, Inc., Cambridge, MA, USA) [16]. The literature selection process was executed in standardized, parallel manner. Two reviewers independently screened the titles and abstracts to filter out potentially eligible studies according to the predefined inclusion and exclusion criteria. Subsequently, a full-text screening was performed. Any disagreements during the selection phases were resolved consulting a third reviewer. PRISMA flowchart illustrating the screening process is shown in Figure 1.

### 2.5. Data Extraction

Data from the included studies were extracted into a pre-structured Microsoft^®^ Excel spreadsheet. The extracted variables comprised: first author, year of publication, geographical location, study design, sample size and characteristics, exposure details, exposure assessment, outcome details, outcomes assessment, key findings, effect estimates alongside their corresponding 95% confidence intervals (CIs), and limits.

### 2.6. Data Synthesis and Statistical Analysis

A qualitative synthesis of the included studies was conducted due to the heterogeneity of the exposures, integrating both narrative and tabular approaches. Studies were organized according to the thematic analysis principles, enabling the identification of recurring patterns across studies. In the first narrative synthesis studies with an impact on osteoporosis-related outcomes were included, while in the second a comparative approach to compare different contaminant classes was chosen.

For ECs classes where data were sufficiently comparable in terms of exposure definitions, target populations, and statistical endpoints, quantitative synthesis was performed. Specifically, studies regarding the long-term impact of fine particulate matter (PM2.5) and coarse particulate matter (PM10) on osteoporosis-related risk were deemed eligible for a meta-analysis because more than three studies were available. The pooled estimates were calculated using a random-effect model via the restricted maximum likelihood (REML) estimation method. This approach explicitly accounts for both within-study sampling error and between-study variance, providing a more conservative and generalizable summary estimate in the presence of clinical or methodological diversity. Effect sizes from individual studies were included in form of Odds Ratios (OR), Relative Risks (RR) and Hazard Ratios (HR) with their 95% CIs. They were analyzed together because of the low prevalence of the considered outcome in included studies. Non-difference line was set at the value of 1. Inter-study heterogeneity was evaluated using Cochran’s Q test (with a significance threshold of *p* < 0.10) and quantified via the I^2^ statistic and τ^2^. To assess the robustness and stability of the pooled summary estimates, iterative leave-one-out sensitivity analyses were conducted by sequentially omitting one study at a time to identify highly influential or outlying data points. Potential publication bias or small-study effects were visually evaluated using funnel plots. All statistical analyses and graphical representations were performed using Stata/MP version 19.5 (StataCorp LLC, College Station, TX, USA, 2026).

### 2.7. Risk-of-Bias Assessment

The methodological quality and internal validity of the included studies were evaluated independently by two investigators using standardized appraisal instruments appropriated to each study design. Cross-sectional studies were appraised with the Joanna Briggs Institute (JBI) Critical Appraisal Checklist [17], a standardized instrument comprising eight key criteria that evaluate participant selection, measurement validity, confounding factors, and statistical analysis. For cohort and case–control designs, the Newcastle–Ottawa Scale (NOS) was employed [18]. It evaluates three essential domains: selection of study groups, comparability of cohorts based on confounding control, and exposure or outcome assessment. Quality assessment was performed by two researchers (V.N. and F.L.) and verified by a third (A.M.). Graphical representations of the bias risk assessment were produced using the robvis R-package [19].

## 3. Results

### 3.1. Overview of Included Studies

At the end of the screening process, a total of 48 studies were analyzed, accounting for 4,510,502 participants. A summary of the included studies is reported in Table 1. Regarding study design, cross-sectional studies predominated (n = 26), followed by cohort studies (n = 12), retrospective cohort studies (n = 4), case–control studies (n = 2), and two-sample Mendelian randomization studies (n = 2), alongside single instances (n = 1 each) of cross-sectional studies with prospective longitudinal mortality follow-up and retrospective time-series studies. Temporally, publication output demonstrated a progressive increase over the decade, distributed across 2016 (n = 2), 2017 (n = 1), 2018 (n = 2), 2019 (n = 1), 2020 (n = 3), 2021 (n = 2), 2022 (n = 5), 2023 (n = 9), 2024 (n = 13), and 2025 (n = 11). Geographically, the United States contributed the largest share (n = 19), followed by China (n = 13), the United Kingdom (n = 5), and South Korea (n = 3), a multicentric collaboration (n = 1), with the remaining articles originating from the Czech Republic, Japan, Mexico, Saudi Arabia, Spain, Sweden, and Taiwan (n = 1 each).

### 3.2. Impact of PM 2.5 Exposure on Osteoporosis and Bone Mineral Density

**Figure 2:**
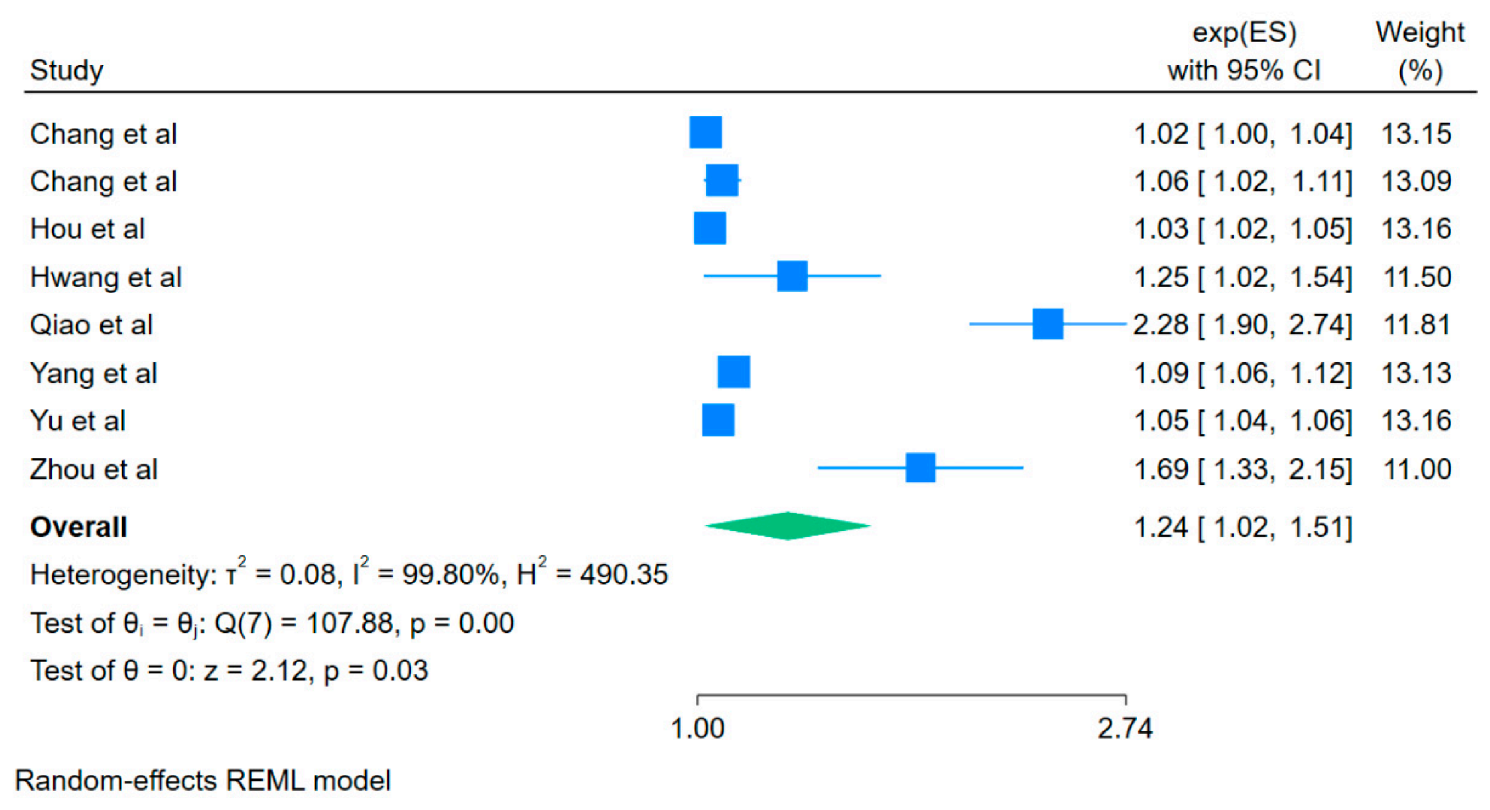
Forest plot of the random-effects meta-analysis evaluating the effects of PM2.5 exposure on osteoporosis [23,24,37,39,48,61,63,67]. For each study, the blue squares and horizontal lines represent the ORs and 95% CIs, with square sizes proportional to study weights. Non-difference line was set at the value of 1. The green diamond indicates the overall pooled effect size (OR = 1.24, 95% CI: 1.02–1.51), which demonstrates a statistically significant increase in risk (*p* = 0.03).

**Figure 3:**
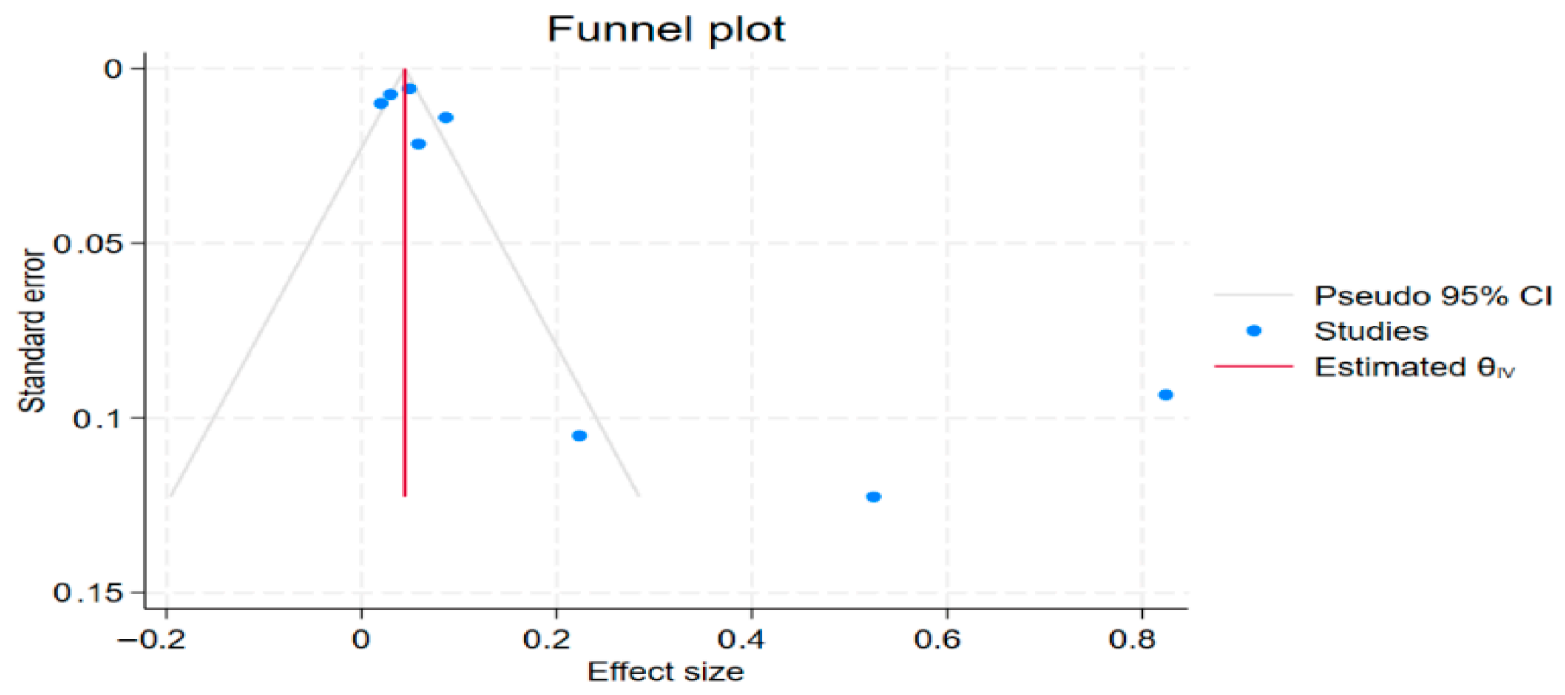
Funnel plot for the assessment of publication bias in the PM2.5 meta-analysis. Individual studies are plotted as blue dots, mapping the effect size against the standard error. The vertical red line indicates the fixed-effects inverse-variance summary estimate, surrounded by the pseudo-95% confidence intervals (diagonal grey lines).

To pool the evidence from the eligible studies regarding impact of PM 2.5 exposure on osteoporosis and BMD, a random-effects meta-analysis REML estimation was executed. The overall effect size indicated a statistically significant risk increase, with a pooled OR = 1.24 (95% CI: 1.02–1.51, *p* = 0.0338). Nevertheless, the evaluation of inter-study variance revealed severe and statistically significant heterogeneity (Q = 107.88, *p* < 0.0001; I^2^ = 99.80%, τ^2^ = 0.0762). This extreme heterogeneity signifies that the magnitude of the effect is highly inconsistent across the analyzed literature, indicating that specific study-level covariates may heavily modulate the primary outcome (Figure 2). The funnel plot evaluation shows a substantial visual asymmetry and the presence of small studies with larger standard errors distributed significantly to the right of the confidence limits, suggesting potential publication bias or small-study effects (Figure 3).

**Figure 4:**
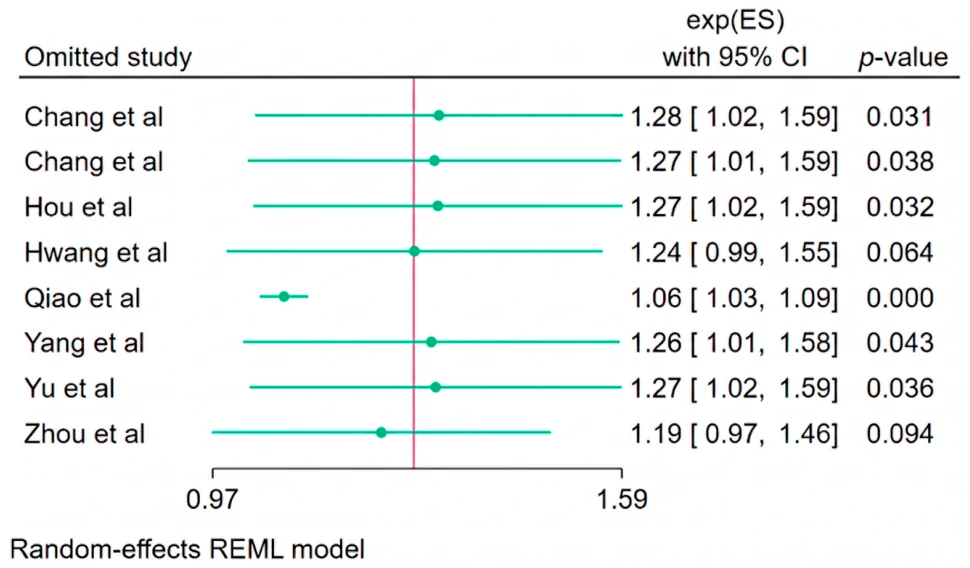
Leave-one-out sensitivity analysis plot evaluating the robustness of the pooled effect size [23,24,37,39,48,61,63,67]. The green dots and horizontal lines represent the recalculated overall ORs and 95% CIs after the iterative omission of each study. The vertical red line indicates the original primary summary estimate.

The leave-one-out sensitivity analysis displays that the overall pooled effect size proved moderately stable, though highly sensitive to specific investigations. Notably, the exclusion of Qiao et al. markedly altered the findings, shifting the summary estimate toward a much narrower and lower effect size (OR = 1.06, 95% CI: 1.03–1.09) while dramatically increasing the statistical precision (*p* < 0.001) [48]. Conversely, the exclusion of either Hwang et al. [39] (OR = 1.24, 95% CI: 0.99–1.55, *p* = 0.064) or Zhou et al. [67] (OR = 1.19, 95% CI: 0.97–1.46, *p* = 0.094) caused the pooled effect size to lose its statistical significance (Figure 4).

### 3.3. Impact of PM 10 Exposure on Osteoporosis and Bone Mineral Density

**Figure 5:**
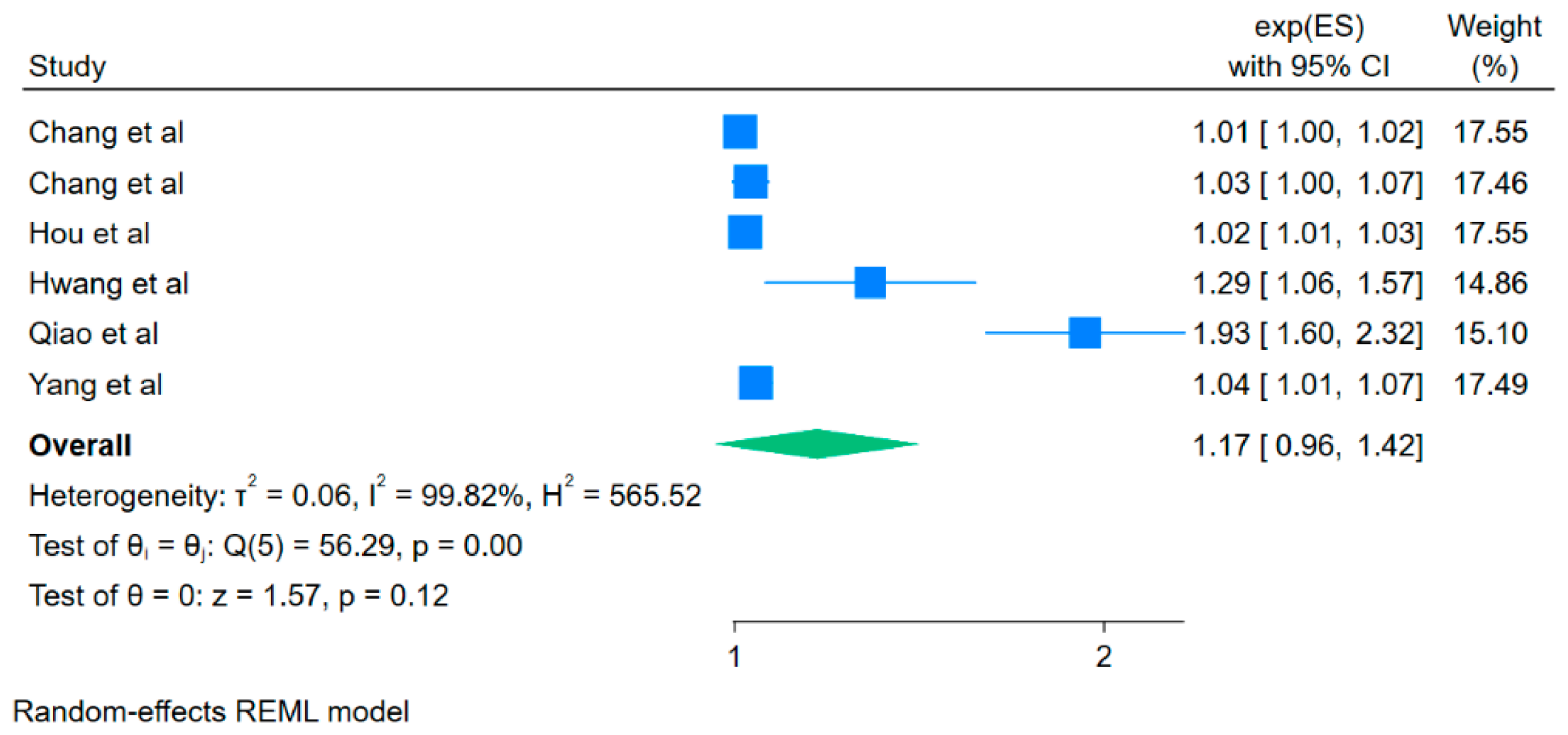
Forest plot of the random-effects meta-analysis evaluating the effects of PM10 exposure [23,24,37,39,48,61]. For each study, the blue squares and horizontal lines represent the ORs and 95% CIs, with square sizes proportional to study weights. Non-difference line was set at the value of 1. The green diamond indicates the overall pooled effect size. Significant and severe heterogeneity was observed across the included studies.

**Figure 6:**
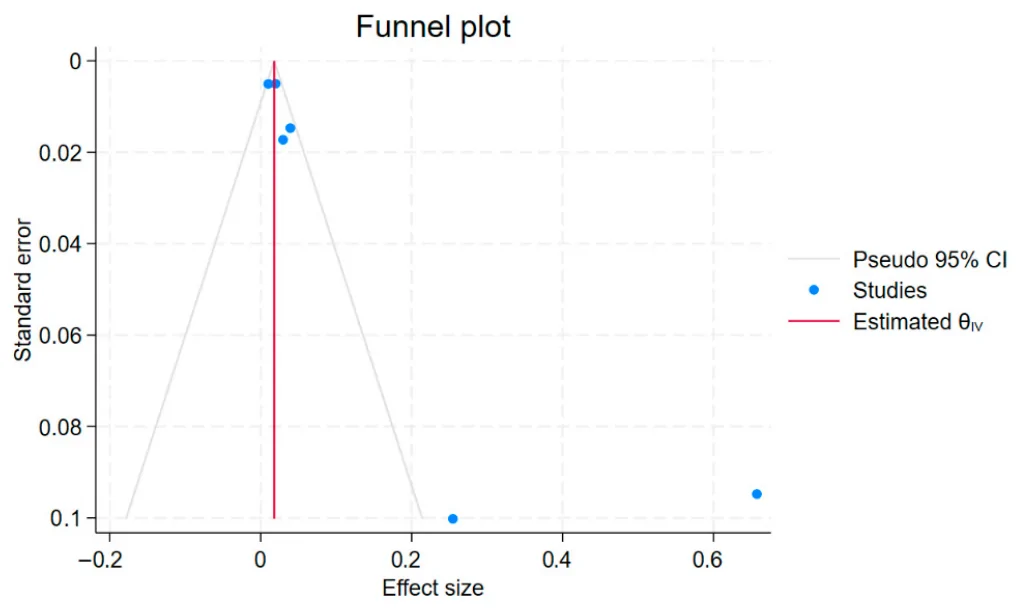
Funnel plot for the PM10 meta-analysis. The blue dots represent individual studies, mapping their respective effect sizes against standard errors. The vertical red line indicates the fixed-effects inverse-variance summary estimate, flanked by the pseudo-95% confidence intervals (diagonal grey lines).

A random-effects meta-analysis was performed to synthesize data from six studies related to PM10 exposure on osteoporosis and BMD. The pooled effect size yielded an overall OR = 1.17 (95% CI: 0.96–1.42, *p* = 0.1170). However, a highly critical and statistically significant level of heterogeneity was detected among the included studies (Q = 56.29, *p* < 0.0001; I^2^ = 99.82%, τ^2^ = 0.0552). This exceptionally high I^2^ value underscores substantial variability across study outcomes, which warrants further investigation through subgroup or sensitivity analyses (Figure 5). At the funnel plot examination, the distribution of the included studies demonstrated marked asymmetry around the overall effect size axis. Specifically, smaller studies with larger standard errors exhibited a pronounced rightward shift outside the pseudo-95% confidence intervals, suggesting an overrepresentation of small studies reporting larger effect sizes. This structural imbalance suggests the potential presence of publication bias or small-study effects within the pooled literature on PM10 exposure (Figure 6).

**Figure 7:**
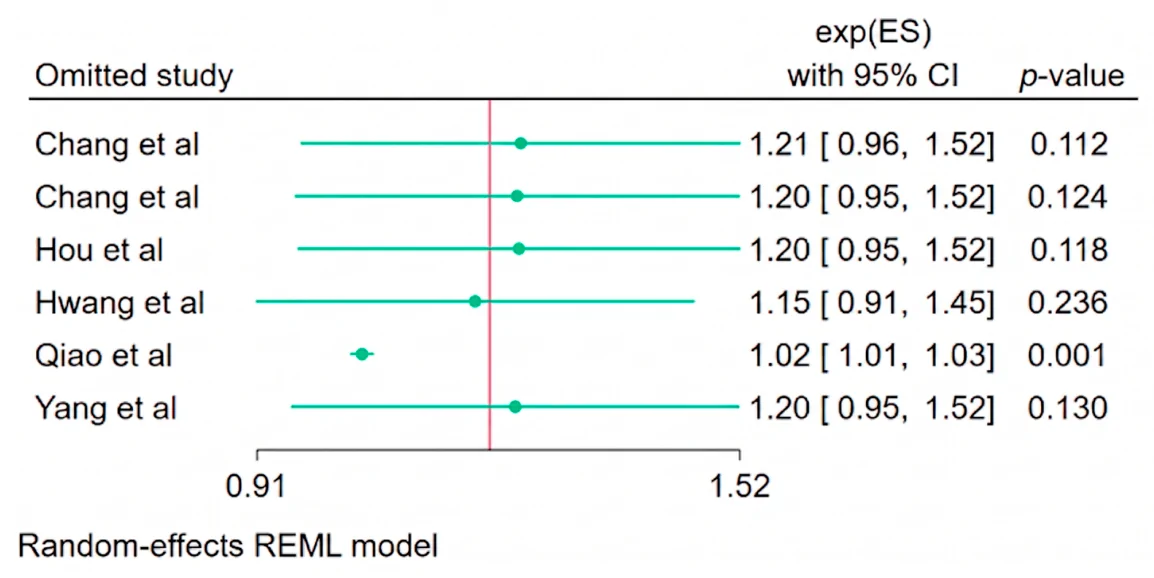
Leave-one-out sensitivity analysis plot evaluating the robustness of the pooled effect size [23,24,37,39,48,61]. The green dots and horizontal lines represent the recalculated overall ORs and 95% CIs after the iterative omission of each study. The vertical red line indicates the original primary summary estimate.

The leave-one-out sensitivity analysis displays that the primary pooled effect size (OR = 1.17, 95% CI: 0.96–1.42, *p* = 0.117) remained non-significant across most iterations, indicating general model stability. However, the analysis revealed that the study by Qiao et al. exerted a disproportionate and statistically confounding influence on the overall model [48]. Upon the specific exclusion of Qiao et al., the pooled estimate shifted significantly toward a lower but statistically significant effect size (OR = 1.02, 95% CI: 1.01–1.03, *p* = 0.001) with a substantial increase in statistical precision (Figure 7) [48].

### 3.4. Impact of Environmental Contaminants on Osteoporosis-Related Outcomes

Overall, the included studies suggest that exposure to ECs may adversely affect skeletal health across different outcome domains, including reduced BMD, increased prevalence or incidence of osteoporosis, impaired bone accrual during growth, and higher risk of osteoporotic fractures. The strongest and most consistent evidence was observed for ambient air pollution. In large population-based cohorts, long-term exposure to fine particulate matter and traffic-related contaminants was repeatedly associated with lower BMD and increased osteoporosis risk. Cheng et al. (2024) reported that a cumulative air pollution score was associated with incident osteoporosis in the UK Biobank, with PM2.5 showing the clearest pollutant-specific association [25]. Xu et al. (2022) similarly found that cumulative air pollution exposure increased the risk of incident osteoporosis, with PM2.5 showing a particularly strong association, while Yang et al. (2023) showed that higher exposure to PM2.5, PM2.5 absorbance, PM10, NO2, and NOx was associated with lower estimated BMD and increased prevalent and incident osteoporosis [58,61]. Yu et al. (2023) extended these findings by demonstrating associations of PM2.5, NO2, and NOx with both osteoporosis and fracture outcomes, also suggesting that genetic susceptibility may modify these associations [63]. In Asian cohorts, Qiao et al. (2020) reported dose–response increases in osteoporosis risk across increasing exposure to PM1, PM2.5, PM10, and NO2 [48]; Shin (2021) found that PM10 was associated with newly diagnosed osteoporosis, particularly among women [51]; Zhang et al. (2022) observed that PM2.5 and SO2 were associated with lower femoral neck t-scores and that PM2.5 increased osteoporosis risk [64]; Chang et al. (2025, Taiwan) found that higher PM2.5 exposure was associated with increased osteoporosis risk and greater t-score decline [23]; Sun et al. (2024) reported associations between long-term exposure to several air contaminants and osteoporosis risk, although some estimates were attenuated after adjustment for environmental and UV-related indicators [53]; and Zhou et al. (2024) showed that PM2.5 and its chemical components were associated with incident osteoporosis, with organic matter showing one of the strongest component-specific associations [67]. Chang et al. (2025, UK) further indicated that black carbon, PM1, and PM2.5 were associated with lower estimated BMD and higher osteoporosis risk [24]. Evidence on fractures was less abundant but clinically important: Heo (2022) found that SO2, but not PM10, CO, NO2, or O3, was associated with osteoporotic fractures in a nationwide Korean cohort, whereas Hou (2024) showed that short-term increases in PM2.5 and PM10 were associated with same-day hospital admissions for osteoporotic fractures, with delayed effects for NO2 and SO2 [36,37]. Two Mendelian randomization studies supported a possible causal contribution of air pollution to skeletal deterioration, as Du et al. (2024) and Ju (2025) both reported inverse associations of genetically predicted PM2.5 and NOx with total-body BMD [28,41].

Beyond air pollution, PFAS represented the second most frequently investigated contaminant group and showed repeated, although not fully consistent, associations with adverse skeletal outcomes. Khalil (2016) found that PFOA, PFHxS, and PFNA were associated with higher odds of osteoporosis in women, while PFOS was associated with lower BMD [42]. Hu (2019) reported that PFOA and PFOS were associated with lower baseline BMD and that PFOS, PFNA, and PFDA predicted greater hip BMD decline over follow-up [38]. Lin (2024) showed that midlife PFAS concentrations were associated with lower lumbar and femoral t-scores, and Wei et al. (2025) reported that PFOA, PFOS, PFHxS, and PFNA were associated with increased osteoporosis risk, with PFAS mixtures associated with both higher osteoporosis risk and lower lumbar BMD [44,56]. Fan et al. (2023) found inverse associations between several legacy and emerging PFAS and BMD t-score, although only PFHpA was significantly associated with osteoporosis [29]. Kirk (2023) showed that PFAS mixture metrics improved prediction of trunk BMD, particularly among women [43]. Evidence from younger populations suggested that PFAS may interfere with bone accrual: Beglarian et al. (2024) found that PFOS was associated with reduced BMD accrual during adolescence and lower total BMD in young adults [22]. Xu et al. (2023) provided clinically relevant evidence that PFAS-contaminated drinking water exposure was associated with major osteoporotic fractures and hip fractures, while Ward-Caviness et al. (2022) linked PFAS exposure in drinking water systems to multimorbidity including osteoporosis-related conditions [55,57]. However, not all PFAS studies were concordant: Banjabi et al. (2020) found that crude associations between PFUnDA, PFOA, PFNA, and osteoporosis were attenuated after adjustment, and Colicino et al. (2020) did not observe statistically significant associations between PFAS mixtures and BMD [21,26].

Several studies also evaluated metals and other emerging or endocrine-disrupting contaminants. For metals, Liu (2024) found that urinary metal mixtures were associated with higher osteoporosis risk, with cadmium emerging as the main harmful contributor, while Peng et al. (2025) reported that blood cadmium was associated with higher odds of osteoporosis and lower femoral BMD, with selenium and manganese showing potentially protective associations [45,46]. India-Aldana (2025) observed pregnancy-related associations between bone-seeking metals and bone strength indices, with cadmium, lead, and manganese showing adverse signals, whereas Puerto-Parejo et al. (2017) did not find significant associations between dietary cadmium, lead, or mercury intake and low BMD in postmenopausal women [40,47]. Among other contaminant classes, Di et al. (2023) showed that mixtures of endocrine-disrupting chemicals, including PAH, pesticide, and phthalate metabolites, were inversely associated with BMD and associated with increased osteoporosis risk in women [27]. Guo et al. (2018) reported that urinary PAH metabolites were associated with lower BMD and higher odds of osteoporosis in women, while Guo (2024) found inverse associations between organophosphate ester metabolites and total-body or lumbar spine BMD in men [34,35]. Shen et al. (2024) showed that pyrethroid metabolites, particularly trans-DCCA, were associated with lower BMD and higher odds of low BMD, whereas Yan et al. (2023) found that associations between trichlorophenols and BMD largely disappeared after adjustment, leaving only a marginal association for 2,4,5-TCP and lumbar BMD [50,59]. For phthalates and phenolic compounds, Reeves et al. (2021) reported that phthalate biomarkers were associated with lower hip and femoral neck BMD and greater hip BMD decline among postmenopausal women not using hormone therapy, Yang et al. (2025, USA) found inverse associations between MECPP and BMD at multiple skeletal sites but paradoxical positive associations for MEHP, and Vitku et al. (2018) reported mostly null findings for bisphenols and parabens in a very small postmenopausal sample, with only exploratory associations involving calcium and bone turnover markers [49,54,60]. VOCs and aldehydes also showed emerging adverse signals: Gu et al. (2023) found that aldehyde mixtures were inversely associated with femoral BMD in men, Fu et al. (2025) reported positive associations between several urinary VOC metabolites and osteoporosis prevalence, and Zhou et al. (2025) found that VOC metabolites and VOC mixtures were associated with higher osteoporosis odds and lower lumbar BMD [30,33,66]. Evidence for POPs and related compounds was more heterogeneous: Fukushi et al. (2016) found that a dioxin-like compound was inversely associated with radial BMD in women [31]; Gao et al. (2024) reported a strong association between non-dioxin-like PCBs and a composite arthritis/osteoporosis outcome [32]; and Bai et al. (2024) found that initial associations between PBDE/PBB compounds and BMD were no longer significant after adjustment for sex [20]. Finally, Sun et al. (2023) provided developmental evidence that blood trihalomethanes, as water disinfection by-products, were associated with lower lumbar and total-body-less-head BMD z-scores in adolescents [52]. Taken together, these findings indicate that ECs may affect bone health through multiple pathways and at different life stages, with the clearest adverse signals involving reduced BMD, increased osteoporosis risk, impaired bone accrual, and, for selected exposures, higher risk of osteoporotic fractures.

### 3.5. Comparative Impact of Different Contaminant Classes on Skeletal Damage

When the included studies are compared by contaminant class, the evidence suggests that skeletal toxicity is not uniform across environmental exposures. Ambient air pollution represents the most consistently investigated and most robustly associated exposure category. Several large cohort studies reported associations between long-term exposure to fine particulate matter or traffic-related contaminants and lower BMD, higher osteoporosis risk, or fracture-related outcomes. In particular, PM2.5 was repeatedly associated with adverse skeletal outcomes in large population-based studies, including those by Cheng et al. (2024), Xu et al. (2022), Yang et al. (2023), Yu et al. (2023), Chang et al. (2025), Zhang et al. (2022), Qiao et al. (2020), and Zhou et al. (2024) [23,25,48,58,61,63,64,67]. Evidence from Mendelian randomization studies by Du et al. (2024) and Ju (2025) further supported a possible causal relationship between PM2.5 or NOx exposure and lower total-body BMD [28,41]. Within this class, finer and combustion-related contaminants, including PM2.5, PM1, black carbon, NO2, and NOx, appeared more consistently associated with bone damage than coarser particulate matter, whereas PM10 and PM2.5–10 showed more heterogeneous results.

PFAS were the second most investigated contaminant class and showed repeated but less uniform associations with skeletal outcomes. Several studies reported adverse associations between specific PFAS and BMD, osteoporosis, or fractures, particularly for PFOA, PFOS, PFHxS, and PFNA. Khalil (2016), Hu (2019), Lin (2024), Fan et al. (2023), Wei et al. (2025), Beglarian et al. (2024), and Xu et al. (2023) collectively suggest that PFAS exposure may affect skeletal health across different life stages, from impaired bone accrual in childhood and adolescence to lower BMD, increased osteoporosis risk, and higher fracture risk in adulthood [22,29,38,42,44,56,57]. However, the evidence was more heterogeneous than for air pollution, as Colicino et al. (2020) did not observe significant associations between PFAS mixtures and BMD, and Banjabi et al. (2020) reported that crude associations with osteoporosis were attenuated after multivariable adjustment [21,26]. Therefore, PFAS can be considered a biologically plausible and repeatedly observed skeletal risk factor, but the current evidence remains less consistent than that available for fine particulate and traffic-related air contaminants.

Among metals, cadmium emerged as the most coherent harmful exposure. Liu (2024) identified cadmium as the main contributor to metal-mixture-related osteoporosis risk [45]^,^, Peng et al. (2025) found that blood cadmium was associated with higher osteoporosis odds and lower femoral BMD, and India-Aldana et al. (2025) reported adverse associations between cadmium exposure and bone strength indices during pregnancy and postpartum [40,46]. Other metals showed more variable findings. Lead, manganese, antimony, nickel, and zinc were associated with adverse skeletal or bone-related comorbidity outcomes in selected studies, whereas selenium, manganese, cobalt, cesium, vanadium, lithium, and strontium showed potentially protective, mixed, or age-dependent associations in other analyses. The null findings reported by Puerto-Parejo et al. (2017) for dietary cadmium, lead, and mercury also suggest that biomonitoring-based measures may better capture skeletal toxicity than dietary intake estimates alone [47].

The evidence for other organic and endocrine-disrupting contaminants was generally more fragmented, but several relevant signals emerged. PAHs, pesticide-related compounds, and organophosphate esters were associated with lower BMD or increased osteoporosis/low BMD risk in Di et al. (2023), Guo et al. (2018), Guo (2024), and Shen et al. (2024), suggesting that these compounds may contribute to bone damage, particularly when evaluated as part of chemical mixtures [27,34,35,50]. By contrast, Yan et al. (2023) found that most associations between trichlorophenols and BMD disappeared after adjustment, indicating weaker evidence for this specific class [59]. Phthalates showed more consistent signals than bisphenols or parabens: Reeves et al. (2021) provided longitudinal evidence linking phthalate biomarkers to BMD decline in postmenopausal women, while Yang et al. (2025) reported inverse associations between MECPP and BMD at multiple skeletal sites [49,60]; conversely, Vitku et al. (2018) reported largely null findings for bisphenols and parabens in a small postmenopausal sample [54]. VOCs and aldehydes should be considered emerging but still preliminary skeletal toxicants, with adverse associations reported by Gu et al. (2023), Fu et al. (2025), and Zhou et al. (2025), although their mostly cross-sectional design and some very large effect estimates require cautious interpretation [30,33,66]. Finally, POPs, BFRs, and water disinfection by-products showed heterogeneous or limited evidence: Fukushi et al. (2016) suggested sex-specific adverse effects of dioxin-like compounds, Gao et al. (2024) reported associations between non-dioxin-like PCBs and a composite arthritis/osteoporosis outcome, Bai et al. (2024) found that PBDE/PBB associations were attenuated after sex adjustment, and Sun et al. (2023) suggested that trihalomethanes may impair adolescent bone accrual [20,31,32,52]. Overall, comparative evidence indicates that fine particulate and traffic-related air contaminants currently represent the most consistent skeletal hazard, followed by selected PFAS and cadmium, whereas PAHs, pesticides, phthalates, VOCs, aldehydes, POPs, brominated flame retardants, and water disinfection by-products provide additional but more preliminary, heterogeneous, or subgroup-specific evidence.

### 3.6. Mechanicistic Profiles and Biochemical Patterns Across Included Studies

**Figure 8:**
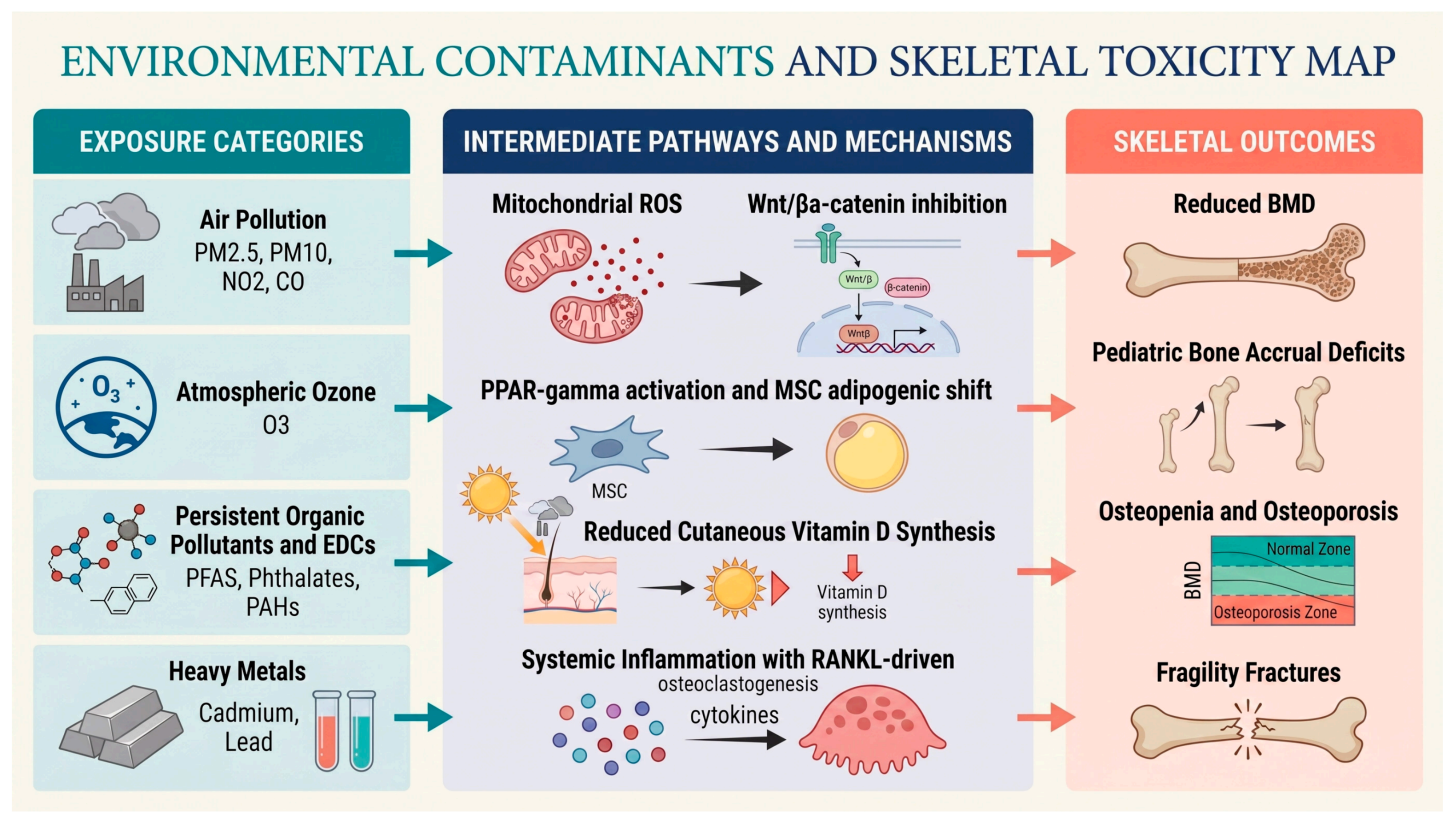
Mechanistic pathways linking environmental contaminants to skeletal toxicity. Environmental exposures impair bone homeostasis via mitochondrial oxidative stress, Wnt/β-catenin downregulation, PPAR-γ-mediated adipogenesis, blunted vitamin D synthesis, and inflammation-driven RANKL osteoclastogenesis, promoting reduced bone mineral density, accrual deficits, osteoporosis, and fragility fractures.

The prospective, cross-sectional, and experimental evidence across 39 distinct studies in this review—comprising investigations on air pollution, PFAS, phthalates, heavy metals, VOCs, and OPEs—reveals a highly convergent, multi-pathway network through which emerging environmental contaminants systematically compromise BMD and accelerate osteoporotic pathology (Figure 8). At the core of this pathophysiological network lies a distinct contrast between the acute, systemic alterations triggered by volatile and gaseous contaminants and the chronic, bioaccumulative damage exerted by POPs and heavy metals. Specifically, criteria air contaminants like PM, NOx, and CO initiate rapid systemic damage, as documented in 11 of the analyzed studies; gaseous contaminants directly bind hemoglobin and cytochrome c oxidase, inducing severe mitochondrial injury, halting oxidative phosphorylation, and generating massive cascades of reactive oxygen species (ROS) that inhibit the osteoprotective Wnt/beta-catenin pathway and activate pro-apoptotic JNK and ERK signaling in osteoblasts and osteocytes. This oxidative microenvironment works in tandem with atmospheric ozone (O3), which, as demonstrated in epidemiological models, uniquely absorbs solar UVB radiation and reduces cutaneous vitamin D synthesis, thereby disrupting the parathyroid–calcium axis and forcing compensatory skeletal resorption. Conversely, persistent contaminants such as PFAS, phthalates, and OPEs—investigated across 12 of the included clinical and cohort studies—alter bone architecture through prolonged endocrine disruption and lineage-specific stem cell reprogramming. These endocrine-disrupting chemicals systematically downregulate estradiol and testosterone—depriving the skeleton of essential anti-apoptotic cues—and competitively antagonize vitamin D receptors to impair calcium absorption. On a cellular level, these organic toxicants, along with bone-seeking heavy metals like Pb and Cd, act as potent agonists of peroxisome proliferator-activated receptor-gamma (PPAR-gamma), which selectively biases mesenchymal stem cell (MSC) differentiation away from bone-forming osteoblasts and toward marrow adipocytes, simultaneously driving bone marrow adiposity and osteopenia. Furthermore, these diverse exposure profiles converge on a chronic, low-grade inflammatory state characterized by elevated neutrophil-to-lymphocyte ratios (NLR) and the sustained release of pro-inflammatory cytokines (TNF-alpha, IL-1beta, IL-6, and IL-17). This inflammatory milieu upregulates the RANKL/OPG ratio, stimulating RANK receptor expression in monocyte-macrophage lineages and accelerating osteoclastogenesis. Finally, at the genomic and structural level, while lead directly displaces calcium within the hydroxyapatite crystal lattice to weaken the cortical bone matrix and cadmium compromises renal calcitriol synthesis, particulate exposure drives accelerated skeletal aging by altering epigenetic age and inducing aberrant DNA methylation across critical osteogenic developmental genes, demonstrating that environmental osteotoxicity represents a complex, interconnected continuum of physical, chemical, and epigenetic degradation.

### 3.7. Risk-of-Bias Assessment

**Figure 9:**
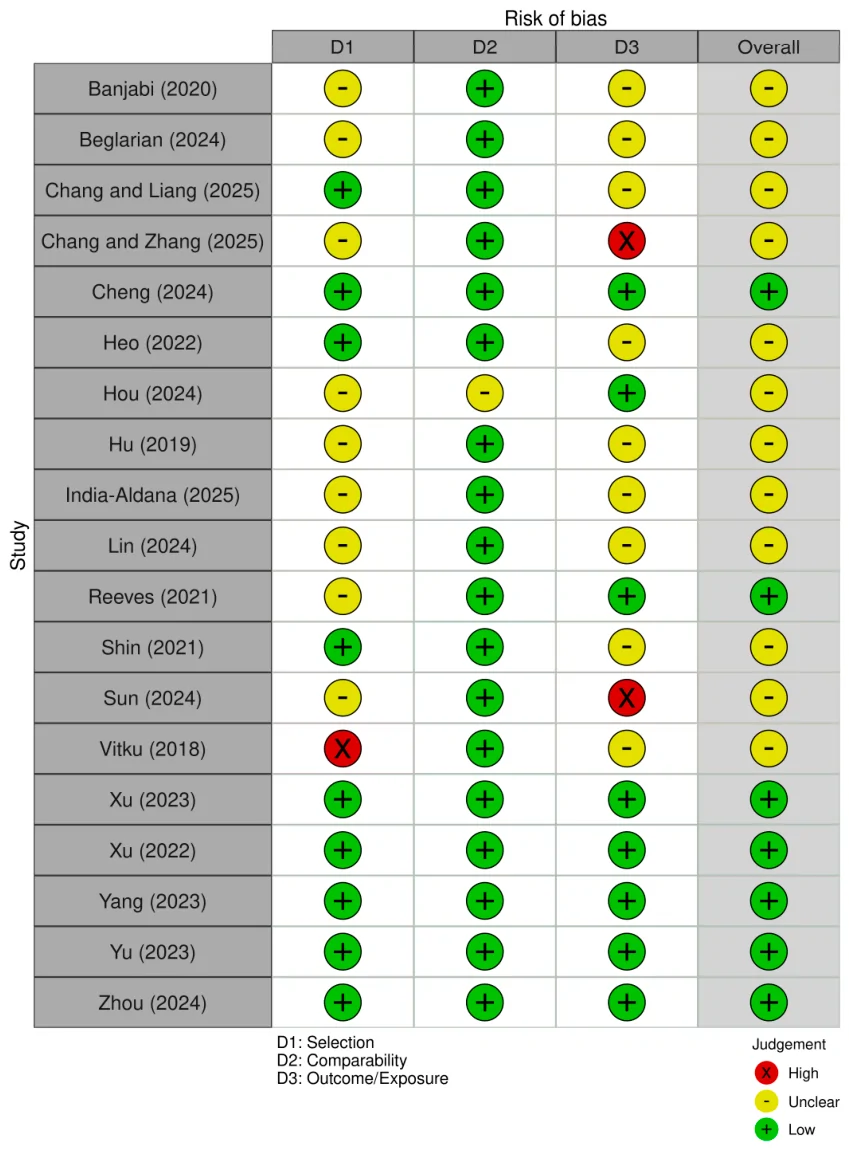
Risk-of-bias summary for the included observational studies evaluated via the Newcastle–Ottawa Scale (NOS), visualized using the *robvis* tool [21,22,23,24,25,36,37,38,40,44,49,51,53,54,57,58,61,63,67]. Judgments are presented for individual domains (D1: Selection, D2: Comparability, D3: Outcome/Exposure) and overall risk of bias for each study. Green (+) indicates low risk, yellow (−) indicates unclear risk (some concerns), and red (X) indicates high risk of bias.

**Figure 10:**
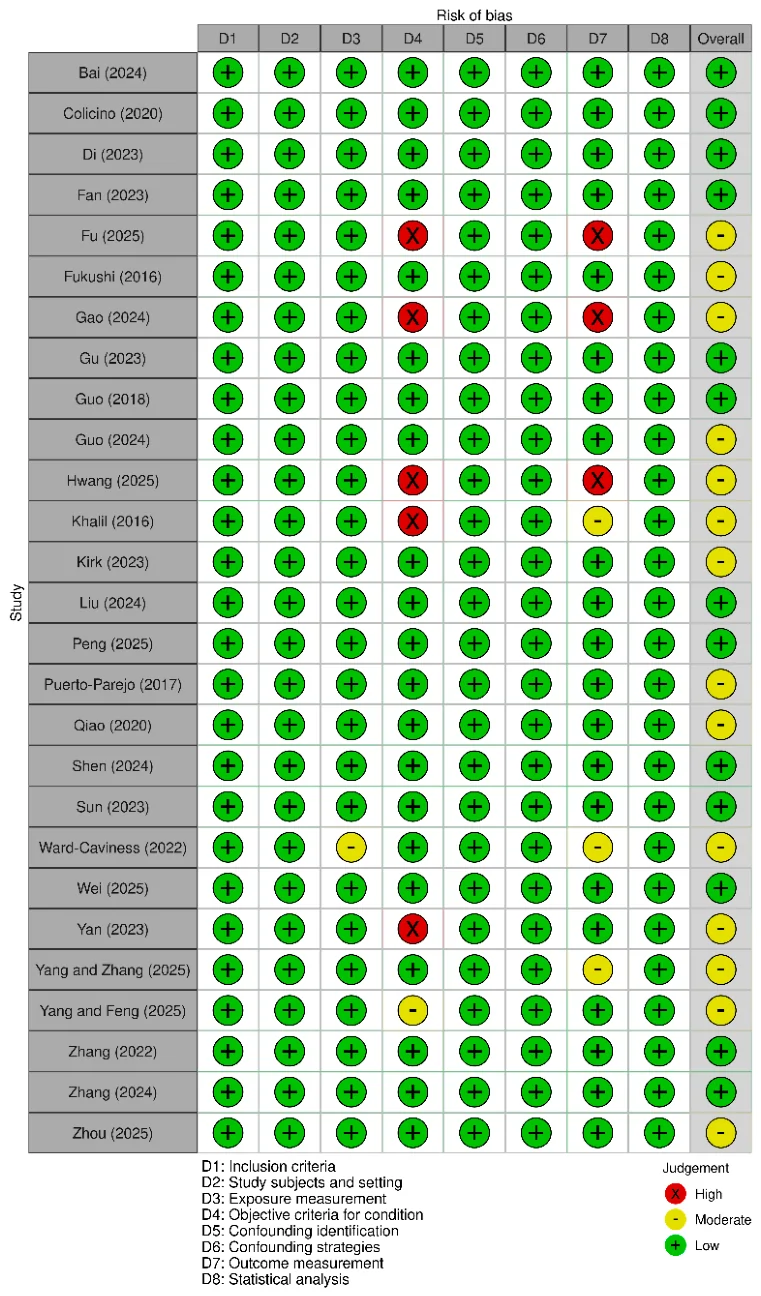
Traffic-light plot presenting the study-level risk of bias assessment for the analytical cross-sectional studies using the Joanna Briggs Institute (JBI) Critical Appraisal Checklist [20,26,27,29,30,31,32,33,34,35,39,42,43,45,46,47,48,50,52,55,56,59,60,62,64,65,66]. D1: Inclusion criteria; D2: Study subjects and setting; D3: Exposure measurement; D4: Objective criteria for condition; D5: Confounding identification; D6: Confounding strategies; D7: Outcome measurement; D8: Statistical analysis. Green (+), yellow (−), and red (X) symbols represent low, moderate, and high risk of bias, respectively.

The risk-of-bias assessment across the included studies demonstrated a predominantly low-to-moderate risk profile. Among the 19 studies evaluated via the NOS scale, 7 demonstrated an overall low risk of bias across all domains, while 12 were classified as having an unclear overall risk due to limitations such as longitudinal attrition and cross-sectional baselines, or specific high-risk domain flags including selection bias in Vítků (2018) [54] and exposure/outcome assessment in Chang and Zhang (2025) [24] and Sun (2024) [53] (Figure 9). For the 27 analytical cross-sectional studies evaluated using the JBI checklist, 13 exhibited an exemplary low risk of bias across all eight domains by employing validated laboratory biomonitoring, gold-standard dual-energy X-ray absorptiometry outcomes, and advanced statistical frameworks to control confounding. In contrast, 14 cross-sectional investigations were graded as having a moderate risk of bias, primarily driven by outcome measurement invalidity from subjective self-reported diagnoses—which introduced high-risk flags for condition criteria and outcome measurements in Fu (2025), Gao (2024), Hwang (2025), and Khalil (2016), and for condition criteria in Yan (2023)—as well as surrogate screening modalities or ecological exposure misclassifications [30,32,39,42,59]. Finally, the studies conducted by Ju et al. and Du et al. could not be evaluated for risk of bias using the employed instruments due to its design as a Mendelian randomization study [28,41] (Figure 10).

## 4. Discussion

The current systematic review and meta-analysis consolidate evidence from forty-eight epidemiological studies, encompassing over 4.5 million participants, to elucidate the complex relationships between exposure to ubiquitous ECs and adverse skeletal outcomes, demonstrating that ambient air contaminants, PFAS, and specific heavy metals are significantly associated with compromised BMD and osteoporosis.

While ambient air pollution exhibits the most robust and consistent epidemiological associations with reduced BMD and incident osteoporosis, the evidence surrounding other endocrine-disrupting chemicals highlights critical, though more heterogeneous, osteotoxic signals.

The exclusive quantitative synthesis focusing on the long-term skeletal impact of particulate matter provides a pivotal yet nuanced understanding of this environmental hazard; specifically, the random-effects meta-analysis revealed a statistically significant positive association between PM2.5 exposure and the risk of osteoporosis, yielding a pooled OR = 1.24 (95% CI: 1.02–1.51). However, the pooled estimate for PM10 exposure, while trending toward an increased risk, failed to reach baseline statistical significance. These summary estimates must be rigorously contextualized by the detection of extreme inter-study heterogeneity, with the I^2^ statistic exceeding 99% for both models, indicating that the magnitude of the osteotoxic effect is highly inconsistent across the global literature, as confirmed by recent systematic reviews [68,69]. This statistical heterogeneity is a reflection of critical exposure, geographic, diagnostic, confounding, and sociodemographic discrepancies across the analyzed cohorts [70].

Regarding the PM2.5 meta-analysis, this variance is primarily driven by disparities in outcome ascertainment and population characteristics across the eight pooled studies, which ranged from peripheral QUS measuring calcaneal estimated bone mineral density (Chang et al., 2025 [24]; Qiao et al., 2020 [48]; Yang et al., 2023 [61]; Yu et al., 2023 [63]) to self-reported physician diagnoses in cancer survivors (Hwang et al., 2025 [39]), hospital admission records for osteoporotic fractures (Hou et al., 2024 [37]), and administrative ICD-10 registry codes capturing clinically advanced osteoporosis (Cheng et al., 2024 [25]; Yang et al., 2023 [61]; Yu et al., 2023 [63]; Zhou et al., 2024 [67]). These diagnostic differences, coupled with non-uniform covariate adjustments for protective lifestyle factors such as physical activity (Chang et al., 2025 [24]) and geographical discrepancies in cohort characteristics across Asian and Western settings, explain why the leave-one-out sensitivity analysis showed high model instability; notably, omitting the large rural QUS cohort by Qiao et al. (2020) [48] markedly compressed the pooled PM2.5 summary estimate from an OR = 1.24 (95% CI: 1.02–1.51) to a highly precise OR = 1.06 (95% CI: 1.03–1.09), whereas omitting Hwang et al. (2025) [39] or Zhou et al. (2024) [67] caused the pooled effect size to lose statistical significance. In parallel, for the PM10 meta-analysis (I^2^ = 99.82%), the six included studies displayed substantial methodological divergence between large prospective biobanks utilizing ICD-10 codes and ultrasound (Chang et al., 2025 [24]; Cheng et al., 2024 [25]; Yang et al., 2023 [61]), fracture hospitalization registries (Hou et al., 2024 [37]), self-reported diagnostic surveys (Hwang et al., 2025 [39]), and cross-sectional rural screening (Qiao et al., 2020 [48]), resulting in an overall baseline estimate of OR = 1.17 (95% CI: 0.96–1.42) that was similarly confounded by the disproportionate weight of Qiao et al. (2020) [48], whose omission shifted the summary estimate to a lower but statistically significant OR = 1.02 (95% CI: 1.01–1.03).

Consequently, while the meta-analysis substantiates the biological plausibility of particulate matter as a skeletal hazard, it concurrently underscores the necessity of moving beyond rudimentary mass-concentration metrics to investigate the specific toxicological components of air pollution driving these signals. From a broader public health and epidemiological standpoint, this heterogeneity aligns with systemic observational challenges across health services research, where descriptive and cross-sectional designs often dominate over prospective, intervention-oriented frameworks, thereby obscuring critical interaction effects and complex vulnerability profiles [71,72,73].

Beyond airborne contaminants, PFAS constitute the second most rigorously evaluated class of ECs, presenting a distinct toxicological profile defined by environmental persistence and profound bioaccumulation within bone and marrow compartments. The synthesized epidemiological evidence reveals that exposure to these substances severely impacts skeletal health across the entire human lifespan, from blunting pediatric bone mineral accrual to accelerating postmenopausal declines in areal BMD and increasing fracture risks in populations chronically exposed to contaminated municipal drinking water. At the cellular level, these osteotoxic effects are fundamentally rooted in the disruption of nuclear receptor signaling; experimental models indicate that PFAS act as potent agonists of the PPAR-gamma pathway within the bone marrow microenvironment, redirecting the lineage commitment of unspecialized mesenchymal stem cells away from osteogenesis and toward an adipogenic fate, resulting in pathological marrow adiposity and structural collapse. Among heavy metals, cadmium consistently emerges as a primary harmful driver within mixture-exposure models. The pathophysiology of cadmium-induced bone disease operates through both direct cellular toxicity, provoking severe mitochondrial damage and premature apoptosis in the osteoblast lineage, and indirect renal impairment, dismantling vitamin D-dependent calcium homeostasis and triggering secondary hyperparathyroidism [74,75].

Furthermore, the synthesized evidence surrounding short-lived endocrine-disrupting chemicals, such as phthalates and bisphenols, suggests biological plausibility for skeletal harm, with specific biomarkers repeatedly linked to lower BMD and altered bone turnover in highly susceptible populations [76,77]. However, the epidemiological evaluation of these short-lived chemicals is perpetually plagued by exposure misclassification, as single-spot urinary biomonitoring provides an exceptionally poor proxy for the long-term, cumulative exposure necessary to induce chronic structural bone diseases, likely underpinning the null or paradoxical non-linear findings frequently reported in cross-sectional datasets.

From a public health and toxicological standpoint, the human skeleton is exposed to complex environmental mixtures rather than isolated contaminants, making the evaluation of cumulative environmental burdens a critical priority. Several included studies addressed this by applying advanced statistical mixture methodologies, such as Weighted Quantile Sum regression, Bayesian Kernel Machine Regression, and Quantile G-computation (Di et al., 2023 [27]; Guo et al., 2024 [34]; Liu et al., 2024 [45]; Wei et al., 2025 [56]). Specifically, WQS models demonstrated that joint exposure to PFAS significantly elevated osteoporosis risk (OR = 1.20; 95% CI: 1.08, 1.32) and reduced lumbar BMD (Wei et al., 2025 [56]), while cumulative urinary metal mixtures similarly increased osteoporosis odds, driven predominantly by cadmium (Liu et al., 2024 [45]). Furthermore, multi-class endocrine-disrupting mixtures (phenols, phthalates, pesticides, and PAHs) were consistently linked to compromised BMD through convergent BKMR and Qgcomp frameworks (Di et al., 2023 [27]), and joint exposures to VOCs (Zhou et al., 2025 [66]) and OPEs (Guo et al., 2024 [35]) further confirmed significant mixture-specific osteotoxicity in adult populations (Guo et al., 2024 [35]; Zhou et al., 2025 [66]). In terms of atmospheric mixtures, joint exposure to air contaminants and chemical components significantly accelerated incident osteoporosis (HR = 1.36; 95% CI: 1.14, 1.61), with organic matter accounting for the maximum weight (Zhou et al., 2024 [67]). These findings have profound public health implications: assessing single contaminants in isolation severely underestimates the true environmental hazard. Integrating mixture-based risk assessments into environmental policies is essential to mitigate the cumulative, real-world osteotoxic burden driving the global rise in metabolic bone diseases.

Although our systematic search protocol captured them, no human observational or interventional studies specifically evaluating micro- and nanoplastics (MNP) exposure in relation to quantitative bone mineral density or fracture risk met our eligibility criteria. MNPs represent pervasive environmental contaminants capable of accumulating across human tissues, including the understudied skeletal compartment [78]. Given that osteoporosis affects over 200 million people worldwide through the disruption of bone remodeling balance, systemic MNP accumulation constitutes a plausible yet critical risk factor for accelerated bone deterioration [78].

Internalization occurs primarily via ingestion and inhalation. Once absorbed, MNPs enter the systemic circulation, and penetrate deep into the bone marrow microenvironment, where inflammation-induced vascular permeability further promotes uptake by hematopoietic progenitor cells [79]. The toxicological profile is strictly size-dependent, with sub-100 nm particles triggering profound intracellular accumulation, sustained autophagy, and Nrf2-mediated oxidative stress, while larger particles provoke acute cytotoxicity [80]. At the cellular level, MNP phagocytosis by bone marrow macrophages induces mitochondrial dysfunction, superoxide production, and NLRP3 inflammasome activation [81]. The ensuing oxidative stress, coupled with osteocyte apoptosis, upregulates RANKL expression without a compensatory rise in OPG, tilting homeostatic signaling toward pathological osteoclastogenesis and bone resorption [81].

Direct epidemiological evidence linking human skeletal pathology to native MNP concentrations remains scarce due to methodological constraints and detection challenges [80]. Nevertheless, extensive biomonitoring of plastic additives confirms consistent negative associations with bone mineral content and skeletal maturation [78]. In addition, translating in vitro dose–response mechanisms to environmentally realistic concentrations continues to present major extrapolation challenges [80].

The skeletal hazard of MNPs is further compounded by environmental co-exposures. PM2.5 and PFAS independently promote osteoclastogenesis, but computational and empirical models demonstrate that nanoplastics actively adsorb PFAS through dispersion forces, facilitating contaminant co-transport and bioaccumulation in deep tissues [82]. Ultimately, simultaneous exposure to MNPs, particulate matter, and PFAS creates potential additive or synergistic toxicities, where cumulative systemic inflammation, targeted contaminant delivery, and pervasive oxidative stress overwhelm the antioxidant capacity of the bone marrow microenvironment [82].

The epidemiological evidence synthesized here demonstrates that environmental contaminants—including PM2.5, heavy metals, and PFAS—act as critical extrinsic drivers of metabolic bone disease yet remain unmeasured in standard clinical algorithms like FRAX. Incorporating residential and occupational exposure histories into routine evaluations, alongside earlier DXA screening, may substantially improve risk stratification for individuals with unexplained osteopenia in highly exposed areas [83].

Skeletal susceptibility is markedly heightened across specific vulnerable demographics. Postmenopausal women exhibit increased sensitivity to endocrine disruptors and air contaminants due to the loss of estrogen-mediated anti-inflammatory protections, while older adults suffer from the cumulative bioaccumulation of bone-seeking toxins coupled with declining antioxidant capacity. These biological vulnerabilities are further compounded by socioeconomic disparities and underlying polygenic risk, emphasizing significant gene–environment interactions in skeletal fragility [84].

To attenuate these hazards, targeted individual and clinical interventions should be prioritized. Regular physical activity stimulates mechanotransductive osteogenesis and mitigates systemic inflammation, while optimal intake of calcium, vitamin D, and antioxidants buffers against competitive uptake and oxidative damage from heavy metals [72]. Complementary exposure-reduction measures, such as indoor HEPA filtration and avoiding contaminated water sources, provide practical risk reduction. Ultimately, regulatory agencies must recognize bone tissue as a primary target of cumulative toxicity, incorporating osteotoxic endpoints into multi-pollutant public health frameworks to curb the growing societal burden of osteoporosis [84].

To the best of our knowledge, this work represents the first systematic review to comprehensively synthesize evidence across the full spectrum of ECs in relation to osteoporosis and BMD while delivering an up-to-date, pollutant-specific meta-analysis on PM2.5 and PM10. Methodological strengths include strict adherence to PRISMA guidelines, dual independent screening, comprehensive search architecture, reliance on large-scale population-based cohorts, and rigorous risk-of-bias appraisals using standardized instruments. Nevertheless, several limitations must be acknowledged. First, critical appraisal of the included literature highlights a prominent dichotomy in methodological quality: while the internal validity of the most robust prospective cohorts is anchored by gold-standard DXA, high-resolution exposure modeling, and thorough adjustment for essential confounders, a substantial proportion of the broader evidence base remains constrained by cross-sectional designs that preclude causal inference and rely on unvalidated, self-reported diagnostic outcomes. Furthermore, the extreme statistical heterogeneity observed across meta-analyzed particulate matter estimates, combined with the difficulty of isolating single-pollutant effects within complex real-world chemical co-exposures, necessitates a cautious interpretation of pooled effect sizes and emphasizes the urgent need for future prospective cohort studies utilizing standardized densitometric measures and advanced mixture-modeling approaches taking into account dose–response modelling. The absence of formal human epidemiological cohorts examining direct MNP exposure on bone health represents a recognized limitation of the current published literature, given that they represent the vast majority of environmental contaminants and constitutes an imperative frontier for prospective environmental epidemiology. Finally, the restriction to English-language publications from 2016 onward carries the inherent risk of language bias and time-window truncation, potentially omitting relevant studies.

## 5. Conclusions

In conclusion, this systematic review and meta-analysis provide comprehensive evidence that exposure to environmental contaminants represents a significant and underrecognized threat to skeletal health, contributing to reduced bone mineral density, impaired bone accrual, and an increased risk of osteoporosis and fractures. Fine particulate matter air pollution, selected per- and polyfluoroalkyl substances, and heavy metals such as cadmium demonstrate the most persistent and robust osteotoxic effects across large observational studies and genetic triangulation models. However, substantial methodological heterogeneity, potential publication bias, and reliance on cross-sectional designs in a significant portion of the literature underscore the necessity for cautious interpretation. Future research must prioritize large-scale, prospective longitudinal cohort studies utilizing standardized densitometric measures, precise biological exposure monitoring, and advanced multi-pollutant mixture modeling to elucidate single-agent and cumulative mechanisms. From a public health perspective, integrating skeletal health monitoring into environmental risk assessments and implementing policies aimed at reducing population-level exposures to airborne and chemical contaminants represent essential strategies for mitigating the global burden of metabolic bone disease.

## Figures and Tables

**Table 1:** Summary of included studies (n = 48) evaluating the association between environmental pollutant exposure, bone mineral density, and osteoporosis risk. Abbreviations: BMD, areal bone mineral density; aOR, adjusted odds ratio; APS, air pollution score; BC, black carbon; BFRs, brominated flame retardants; BKMR, Bayesian kernel machine regression; BMI, body mass index; BWQS, Bayesian weighted quantile sum regression; CHS, Children’s Health Study; Cl-THMs, chlorinated trihalomethanes; CO, carbon monoxide; CTx, C-terminal telopeptide of type I collagen; DXA, dual-energy X-ray absorptiometry; eBMD, estimated bone mineral density; EDCs, endocrine-disrupting chemicals; FN-BMD, femoral neck bone mineral density; GRS, genetic risk score; HR, hazard ratio; HT, hormone therapy; ICD-9/ICD-10, International Classification of Diseases (Ninth and Tenth Revisions); IQR, interquartile range; IVW, inverse-variance weighted; L1-BMD, first lumbar vertebra bone mineral density; MOF, major osteoporotic fracture; ND, not determined; NO2, nitrogen dioxide; NOx, nitrogen oxides; O3, ozone; OH-PAH, monohydroxylated polycyclic aromatic hydrocarbons; OM, organic matter; OP, osteoporosis; OPEs, organophosphate esters; PAEs, phthalate esters; PAHs, polycyclic aromatic hydrocarbons; PAM, Partitioning Around Medoids; PBDEs, polybrominated diphenyl ethers; PCBs, polychlorinated biphenyls; PFASs, per- and polyfluoroalkyl substances; PM1, particulate matter ≤ 1 µm; PM2.5, particulate matter ≤ 2.5 µm; PM2.5–10, coarse particulate matter between 2.5 and 10 µm; PM10, particulate matter ≤ 10 µm; PRS, polygenic risk score; Qgcomp, quantile g-computation; QUS, quantitative ultrasound; RERI, relative excess risk due to interaction; RR, relative risk; SD, standard deviation; SO2, sulfur dioxide; SOLAR, Study of Latino Adolescents at Risk; sub-HR, subdistribution hazard ratio; TBLH, total body less head; TBMD, total bone mineral density; TF-BMD, total femur bone mineral density; THMs/TTHMs, trihalomethanes/total trihalomethanes; TS-BMD, total spine bone mineral density; U-PAHs, urinary polycyclic aromatic hydrocarbons; UV, ultraviolet; VOCs/VOCM, volatile organic compounds/volatile organic compound metabolites; WBGT, wet-bulb globe temperature; WQS, weighted quantile sum.

Author	Year	Country	Study Design	Sample Size	Exposure Assessment	Exposure	Outcome Assessment Method	Key Findings
Bai et al. [20]	2024	United States	Cross-sectional study	3079	Serum biomonitoring	BFRs (PBDEs, PBB153)	DXA	**PBDE-153:** Positively associated with TF-BMD (β = 0.0201, 95% CI: 0.0099, 0.0302), FN-BMD (β = 0.0111, 95% CI: 0.0024, 0.0198), and L1-BMD (β = 0.015, 95% CI: 0.006, 0.0239). **PBB-153:** Positively associated with TF-BMD (β = 0.0177), FN-BMD (β = 0.009), TS-BMD (β = 0.0081), and L1-BMD (β = 0.0144). **Modifiers:** Significance attenuated after sex adjustment; joint BFR mixtures inversely predicted BMD in men.
Banjabi et al. [21]	2020	Saudi Arabia	Case–control study	208	Serum biomonitoring	PFASs	DXA	**Crude Models:** Increased osteoporosis odds for PFUnDA (OR = 25.0, 96.0, 95% CI: 5.47, 464), PFOA (OR = 2.63), and PFNA (OR = 2.54); reduced odds for PFPeA. **Adjusted Models:** All PFAS-osteoporosis associations lost statistical significance after adjusting for gender, age, calcium, vitamin D, fractures, and thyroid.
Beglarian et al. [22]	2024	United States	Cohort study	441	Plasma biomonitoring	PFAS (PFOS, PFOA, PFNA, PFDA, PFHxS)	DXA	**SOLAR Cohort:** Baseline PFOS significantly reduced longitudinal trunk BMD accrual; non-significant negative trajectory for total BMD. PFAS mixtures suppressed annual total BMD change in males (PFOS and PFOA reduced trunk BMD). **CHS Cohort:** Baseline PFOS inversely correlated with total BMD; PFDA negatively correlated with total BMD in females.
Chang et al. [23]	2025	Taiwan	Cohort study	19,981	Spatiotemporal environmental modeling	PM2.5	QUS	**PM2.5:** High exposure increased osteoporosis risk (Q4 vs. Q1 HR = 1.66, 95% CI: 1.43, 1.92, *p* < 0.001; log-transformed HR = 1.73, 95% CI: 1.02, 2.95, *p* = 0.043) and reduced BMD t-scores (β = −0.020, 95% CI: −0.029, −0.005, *p* = 0.004). **WBGT:** Independently increased osteoporosis risk (HR = 1.49, 95% CI: 1.33, 1.66, *p* < 0.001) and amplified risk at low PM2.5 (Q1 HR = 2.93, 95% CI: 2.21, 3.89, *p* < 0.001).
Chang et al. [24]	2025	United Kingdom	Cohort study	233,184	Spatiotemporal environmental modeling	BC, PM1, PM2.5, PM10	QUS	**Pollutant Effects (eBMD & Osteoporosis):** - BC: β = −2.35 × 10^−3^ (*p* = 1.78 × 10^−2^); OR = 1.30 (95% CI: 1.15, 1.48, *p* < 0.001). - PM1: β = −1.57 × 10^−3^ (*p* = 9.04 × 10^−4^); OR = 1.10 (95% CI: 1.04, 1.17, *p* < 0.001). - PM2.5: β = −9.38 × 10^−4^ (*p* = 2.98 × 10^−13^); OR = 1.02 (95% CI: 1.00, 1.04, *p* = 0.019). **Mitigation:** Moderate (OR = 0.69) and high (OR = 0.65) physical activity mitigated risks.
Cheng et al. [25]	2024	United Kingdom	Cohort study	271,321	Spatiotemporal environmental modeling	PM 2.5, PM 2.5–10, NO2, NOx	ICD-10 codes	**Cumulative Score:** Increased osteoporosis risk (HR = 1.07, 95% CI: 1.00, 1.01), driven most strongly by PM2.5 (HR = 1.06, 95% CI: 1.02, 1.11). **Subpopulations:** PM2.5–10 increased risk specifically in normal/underweight individuals; higher vulnerability in females and low socioeconomic status.
Colicino et al. [26]	2020	United States	Cross-sectional study	499	Serum biomonitoring	8 PFASs	DXA	**BWQS Regression (PFAS Mixture):** No significant associations with BMD at lumbar spine (β = −0.004, 95% CrI: −0.04, 0.04), total femur (β = 0.002, 95% CI: −0.04, 0.05), or femur neck (β = 0.005, 95% CrI: −0.03, 0.04) in adults, men > 50, or postmenopausal women. **Single Congeners:** Sm-PFOS and PFNA lost significance after multiple testing correction.
Di et al. [27]	2023	China	Cross-sectional study	6766	Urinary biomonitoring	Phenols, chlorophenol pesticides, phthalates, PAHs	DXA	**EDC Mixture (WQS):** Reduced total femur (β = −0.028 g/cm^2^, 95% CI: −0.040, −0.017), femoral neck (β = −0.015 g/cm^2^, 95% CI: −0.025, −0.004), and L1 BMD (β = −0.018 g/cm^2^, 95% CI: −0.033, −0.003). MnBP had highest weight for total femur BMD reduction. **Single Compounds & Modeling:** Qgcomp confirmed inverse L1-BMD; BKMR linked mixture to female osteoporosis risk. Ln-2-fluorene increased female osteoporosis risk (OR = 1.29, 95% CI: 1.01, 1.64); non-linear effects for specific phthalates.
Du et al. [28]	2024	Europe	Two-sample Mendelian randomization study	423,796	Two-sample Mendelian randomization	PM2.5, PM2.5–10, PM10, NO2, NOx	DXA	**IVW Mendelian Randomization:** Causal links to decreased total-body BMD for genetic liability to nitrogen oxides (β = −0.55, 95% CI: −0.90 to −0.21, *p* = 0.002) and PM2.5 (β = −0.33, 95% CI: −0.59 to −0.08, *p* = 0.010). **Null Associations:** No causal link for PM2.5–10, PM10, or NO2.
Fan et al. [29]	2023	China	Cross-sectional study	1260	Serum biomonitoring	32 PFASs and alternatives	QUS	**11 PFASs:** Inversely associated with BMD t-scores (β range: −0.06 to −0.23 per ln-unit, *p* < 0.05). **PFHpA:** Elevated osteoporosis odds (OR = 1.23, 95% CI: 1.04, 1.45). **Demographics:** Adverse associations strictly restricted to women and younger individuals (<60 years).
Fu et al. [30]	2025	China	Cross-sectional study	14,945	Urinary biomonitoring	15 VOCs	Self-reported physician diagnosis	**Urinary VOCs (Q4):** Elevated osteoporosis risk for CEMA (OR = 32.38, 95% CI: 26.32, 39.84, *p* < 0.001), 3HPMA (OR = 254.02, 95% CI: 149.16, 432.58, *p* < 0.001), and 2MHA (OR = 4.88, 95% CI: 4.43, 5.37, *p* < 0.001). **Null Compounds:** AAMA, CYMA, and ATCA showed no significance. Results consistent across age, sex, and non-smokers.
Fukushi et al. [31]	2016	Japan	Cross-sectional study	489	Serum biomonitoring	Dioxin-related compounds	DXA	**Females:** 1,2,3,4,6,7,8-HpCDD negatively associated with BMD z-scores (β = −1.123, 95% CI: −1.974 to −0.272, *p* = 0.010); other congeners were non-significant. **Males:** Positive associations between specific congeners and BMD z-scores disappeared after adjusting for BMI.
Gao et al. [32]	2024	United States	Cross-sectional study with prospective longitudinal mortality follow-up	10,961	Serum biomonitoring	7 PCBs	ND	**PCBs:** Positive correlation with composite arthritis and osteoporosis prevalence (OR = 6.27, 95% CI: 5.23, 7.55, *p* < 0.0001).
Gu et al. [33]	2023	United States	Cross-sectional study	1039	Serum biomonitoring	Aldehydes	DXA	**Qgcomp/BKMR:** Aldehyde mixture negatively associated with femoral BMD in men (not women). **Males:** Hexanaldehyde associated with total femur (β = −0.07), intertrochanter (β = −0.10), and lumbar spine BMD (β = −0.09). Propanaldehyde was destructive at ≤3.80 ng/mL (β = −0.09) and protective at >3.80 ng/mL (β = 0.39). Isopentanaldehyde > 1.05 ng/mL was osteoprotective (β = 0.22).
Guo et al. [34]	2024	United States	Cross-sectional study	3546	Urinary biomonitoring	5 OPEs	DXA	**Single OPEs in Men:** BCPP linked to total-body BMD (β = −0.013, 95% CI: −0.026, −0.001); DBUP (β = −0.022) and BCEP (β = −0.018) linked to lumbar spine BMD. **OPE Mixture:** Significantly reduced total-body and lumbar spine BMD in men (75th vs. 25th percentile: −0.018 g/cm^2^), highest in age ≥ 50. No significant associations in females.
Guo et al. [35]	2018	United States	Cross-sectional study	1768	Urinary biomonitoring	PAHs	DXA	**Adult Women (U-PAHs T3):** Reduced total femur BMD for 2-hydroxyfluorene (95% CI: −0.028, −0.001) and 2-hydroxyphenanthrene (95% CI: −0.033, −0.007). **Osteoporosis Odds:** 3-hydroxyphenanthrene total femur OR = 3.09 (95% CI: 1.51, 6.34); overall OR = 2.28. Strongest postmenopausally; absent in men.
Heo et al. [36]	2022	South Korea	Retrospective cohort study	56,467	Spatiotemporal environmental modeling	PM10, SO2, CO, NO2, O3, PM2.5	ICD-10 and procedure codes	**SO2 (3-Year Moving Average):** Elevated incident osteoporotic fractures (HR = 1.04, 95% CI: 1.00, 1.09 per 2 ppb IQR increase). **Null Exposures:** PM10, CO, NO2, and O3 were not significantly associated with fractures.
Hou et al. [37]	2024	China	Retrospective time-series study	18,933	Spatiotemporal environmental modeling	PM2.5, PM10 and gaseous contaminants (SO2, CO, NO2, and O3)	Hospital admission diagnoses	**Fracture Hospitalizations (per 10 µg/m^3^):** Peaked at lag 0 for PM2.5 (RR = 1.03, 95% CI: 1.02, 1.04) and PM10 (RR = 1.02, 95% CI: 1.01, 1.02); peaked at lag 11 for SO2 (RR = 1.05, 95% CI: 1.03, 1.07) and NO2 (RR = 1.06, 95% CI: 1.04–1.07). **Sex Specificity:** SO2 and NO2 effects were strictly significant in women.
Hu et al. [38]	2019	United States	Cohort study	294	Plasma biomonitoring	PFOS, PFOA, PFHxS, PFNA, PFDA	DXA	**Cross-Sectional (Baseline per SD):** PFOS (β = −0.020, 95% CI: −0.037 to −0.003) and PFOA (β = −0.021) inversely associated with spine BMD. **Longitudinal (2-Year Decline per SD):** Accelerated total hip BMD loss for baseline PFOS (β = −0.005, 95% CI: −0.009 to −0.001), PFNA (β = −0.006), and PFDA (β = −0.005).
Hwang et al. [39]	2025	South Korea	Cross-sectional study	8977	Spatiotemporal environmental modeling	PM10, PM2.5, and gaseous contaminants (SO2, NO2, and CO)	Self-reported current status based on previous physician diagnosis	**Cancer Survivors (3-Year Exposure):** PM10 (OR = 1.20, 95% CI: 1.00, 1.43 per IQR) and SO2 (OR = 1.16, 95% CI: 1.01, 1.32) elevated osteoporosis odds. **Female Cancer Survivors (1-Year Exposure):** Elevated risk for PM2.5 (OR = 1.25, 95% CI: 1.02, 1.54), NO2 (OR = 1.42, 95% CI: 1.06, 1.90), and PM10 (OR = 1.29, 95% CI: 1.06, 1.57). Non-significant in cancer-free controls and males.
India-Aldana et al. [40]	2025	Mexico	Cohort study	599	Blood and urinary biomonitoring	Al, Ba, Cd, Mn, Pb	QUS	**Radius Z-Scores:** Positively associated with Al (β = 0.10, 95% CI: 0.02, 0.18); postpartum Cd reduced scores (β = −0.30, 95% CI: −0.49, −0.11). **Phalanx Z-Scores:** Decreased with Mn (β = −0.10, 95% CI: −0.18, −0.03), Pb (β = −0.09, 95% CI: −0.16, −0.02), and Al (β = −0.12, 95% CI: −0.21, −0.03).
Ju et al. [41]	2025	China	Two-sample Mendelian randomization study	Exposure dataset: 1,791,870 participants Outcome dataset: 56,284 participants	Two-sample Mendelian randomization	NO2, NOx, PM2.5, PM10	Al, Ba, Cd, Mn, Pb	**IVW Mendelian Randomization:** Causal link between genetically predicted total-body BMD loss and nitrogen oxides (β = −0.59, 95% CI: −1.03 to −0.16, *p* = 0.008) as well as PM2.5 (β = −0.60, 95% CI: −1.12 to −0.08, *p* = 0.025).
Khalil et al. [42]	2016	United States	Cross-sectional study	1914	Serum biomonitoring	PFOA, PFOS, PFHxS, PFNA	DXA and self-reported diagnosis	**Females (Osteoporosis Odds):** ln-PFOA (aOR = 1.84, 95% CI: 1.17, 2.90; Q4 vs. Q1 aOR = 2.59), PFHxS (aOR = 1.64; Q4 vs. Q1 aOR = 13.20), and PFNA (aOR = 1.45; Q4 vs. Q1 aOR = 3.23). **Males (FNBMD):** ln-PFOS inversely associated (β = −0.013, 95% CI: −0.024, −0.002; Q4 vs. Q1 β = −0.046).
Kirk et al. [43]	2023	United States	Cross-sectional study	1004	Serum biomonitoring	PFAS (PFDA, PFHxS, PFNA, PFUA, n-PFOS, Me-PFOSA-AcOH)	DXA	**PFAS Mixture on TBMD:** Improved approximation by 4.7% in the combined cohort. **Sexual Dimorphism:** Improved approximation by 11.1% in females vs. 3.2% in males (relative effect 3.4 times greater in females).
Lin et al. [44]	2024	United States	Cohort study	531	Plasma biomonitoring	PFAS Six specific PFAS were evaluated during pregnancy (PFOS, PFOA, PFHxS, PFNA, MeFOSAA, EtFOSAA) and six were evaluated at midlife (PFOS, PFOA, PFHxS, PFNA, PFDA, PFUnDA)	DXA	**Midlife PFAS:** Concurrent aBMD reduced per PFOA doubling (lumbar spine t-score: −0.21, 95% CI: −0.35, −0.07), per PFAS IQR increment (spine t-score: −0.18, 95% CI: −0.33, −0.04), and in postmenopausal women (spine t-score: −0.40). **Pregnancy PFAS:** PFOA doubling prospectively associated with higher midlife spine t-score (0.27, 95% CI: 0.07, 0.48).
Liu et al. [45]	2024	China	Cross-sectional study	15,923	Urinary biomonitoring	Ba, Cd, Co, Cs, Mo, Pb, Sb, Tl, Tu	DXA	**Metal Mixture (WQS Doubling):** Increased osteoporosis odds (OR = 1.19), driven by urinary Cd (weight = 0.66; OR = 1.19, 95% CI: 1.08, 1.31), Sb, Pb, and Tu. **PAM Clustering:** High-exposure cluster had a 1.74-fold higher osteoporosis risk (OR = 1.74, 95% CI: 1.43, 2.12).
Peng et al. [46]	2025	United States	Cross-sectional study	905	Blood biomonitoring	Pb, Cd, Hg, Se, Mn	DXA	**Cadmium (Cd):** High blood Cd (Q4) doubled OP risk (OR = 2.18, 95% CI: 1.31, 3.63) and lowered total femur BMD (β = −0.112, *p* = 0.007). Combined metals (>50th percentile) elevated OP risk. **Selenium (Se) & Manganese (Mn):** Se Q4 was protective (OR = 0.52, 95% CI: 0.31, 0.87); Mn positively correlated with total femur (β = 0.072) and femoral neck BMD (β = 0.082).
Puerto-Parejo et al. [47]	2017	Spain	Cross-sectional study	281	Validated dietary assessment	Cd, Pb, Hg	QUS and DXA	**Dietary Intake (Cd, Pb, Hg):** No significant association with low hip/lumbar BMD (high Cd spine OR = 0.767, 95% CI: 0.396–1.489; high Hg hip OR = 1.458, 95% CI: 0.557–3.818). **Combined Metals:** High intake of all three metals showed slightly higher lumbar spine areal BMD (*p* = 0.037).
Qiao et al. [48]	2020	China	Cross-sectional study	8033	Spatiotemporal environmental modeling	PM1, PM2.5, PM10, NO2	QUS	**Continuous Exposure (Q4 vs. Q1 OP OR):** PM1 = 2.08 (95% CI: 1.72, 2.50), PM2.5 = 2.28 (95% CI: 1.90, 2.74), PM10 = 1.93 (95% CI: 1.60, 2.32), NO2 = 2.02 (95% CI: 1.68, 2.41). **Per SD Increase in OP Risk:** PM1 +28.5%, PM2.5 +29.6%, PM10 +32.4%, NO2 +29.7%. High physical activity mitigated risk.
Reeves et al. [49]	2021	United States	Cohort study	1255	Urinary biomonitoring	13 phthalate biomarkers	DXA	**Non-HT Users:** MCPP Q4 linked to lower total hip (β = −0.0069 g/cm^2^) and femoral neck BMD (β = −0.00737 g/cm^2^); DiBP Q4 linked to lower total hip BMD (β = −11.09 × 10^−3^ g/cm^2^); high MCOP and MCNP accelerated 3-year total hip decline (−1.80% and −1.84%). **Hormone Therapy (HT):** HT completely neutralized these adverse effects.
Shen et al. [50]	2024	United States	Cross-sectional study	1389	Urinary biomonitoring	Pyrethroid metabolites (3-PBA, trans-DCCA, 4-F-3PBA)	13 phthalate biomarkers	**Trans-DCCA:** Tertile 2 reduced total spine BMD (β = −0.041, 95% CI: −0.078, −0.004) and increased low BMD risk (tertile 2 OR = 1.63, 95% CI: 1.07, 2.48; tertile 3 OR = 1.65, 95% CI: 1.10, 2.50). Male tertile 3 OR = 1.88 (95% CI: 1.01, 3.51). **Pyrethroid Mixture:** Elevated low BMD risk (OR = 1.13, 95% CI: 1.01, 1.27), driven by 3-PBA.
Shin et al. [51]	2021	South Korea	Retrospective cohort study	237,149	Spatiotemporal environmental modeling	PM10, PM2.5, NO2, CO, SO2	ICD-10 codes	**PM10 Exposure:** 22.2% diagnosed with osteoporosis. Q4 vs. Q1 adjusted HR = 1.03 (95% CI: 1.01–1.06). **Subgroups:** Females (sub-HR = 1.07, 95% CI: 1.00–1.13), age < 65 (sub-HR = 1.04, 95% CI: 1.01–1.07), low-urbanization (sub-HR = 1.05, 95% CI: 1.02–1.09). NO2, SO2, CO, PM2.5 were non-significant.
Sun et al. [52]	2023	United States	Cross-sectional study	Lumbar spine BMD: 2294 participants Total body less head: 1350 participants Total: 3644 participants	Blood biomonitoring and tap water analysis	THMs (TCM, BDCM, DBCM, TBM)	DXA	**Blood THMs (2.7-Fold Increase):** - Spine BMD z-scores: TCM (β = −0.06), DBCM (β = −0.06), Cl-THMs (β = −0.08), TTHMs (β = −0.07). - TBLH BMD z-scores: BDCM (β = −0.10), DBCM (β = −0.10), Cl-THMs (β = −0.11). **Modifier:** Cl-THM effects stronger in overweight/obese adolescents (*p*-interaction = 0.005).
Sun et al. [53]	2024	China	Retrospective cohort study	2361	Spatiotemporal environmental modeling	PM1, PM2.5, PM10, SO2, NO2, CO, O3	DXA	**5-Year Exposure (OP Risk):** PM1 (+9.5% per 1 µg/m^3^), PM2.5 (+5.4% per 1 µg/m^3^). Linear dose–response for PM1, PM2.5, PM10, NO2; O3 and UV were protective. **Subgroups:** PM1/PM2.5 risks stronger in males (*p* = 0.02), BMI ≥ 25 kg/m^2^, and age ≥ 60. SO2 and CO were non-significant.
Vitku et al. [54]	2018	Czech Republic	Case–control study	24	Plasma biomonitoring	Bisphenols, parabens	DXA	**Cases vs. Controls:** No significant differences in plasma BPA or methyl paraben (MP). BPS, BPF, and BPAF were below detection limits. **Markers:** BPA positively associated with total plasma calcium (β = 0.077, *p* = 0.033); MP inversely associated with CTx (β = −0.232, *p* = 0.0279).
Ward-Caviness et al. [55]	2022	United States	Cross-sectional study	10,168	Spatiotemporal environmental modeling	PFAS (PFOA, PFHpA, PFOS, PFHxS)	ICD-9 and ICD-10 billing codes	**Any PFAS Exposure:** Elevated odds for multimorbidity (OR = 1.25, 95% CI: 1.09, 1.45), one extra chronic condition (OR = 1.24, 95% CI: 1.10, 1.39), and osteoporosis (OR = 1.45, 95% CI: 1.05, 2.01). **Specific Exposures:** PFOA multimorbidity OR = 1.30 (95% CI: 1.12, 1.52); joint PFOA + PFHpA multimorbidity OR = 1.38 (95% CI: 1.09, 1.76 vs. single OR = 1.24). Stronger in White and low-income groups; no sex differences.
Wei et al. [56]	2025	United States	Cross-sectional study	2764	Serum biomonitoring	PFOA, PFOS, PFHxS, PFDeA, PFNA	DXA	**Single PFAS (per ln-unit OP OR):** PFOA (OR = 1.96, 95% CI: 1.43, 2.69), PFOS (OR = 1.42, 95% CI: 1.06, 1.91), PFHxS (OR = 1.53, 95% CI: 1.14, 2.05), PFNA (OR = 1.60, 95% CI: 1.22, 2.10). **BMD & Mixtures:** Lumbar BMD reduced by ln-PFOS (β = −0.019 g/cm^2^) and ln-PFHxS (β = −0.014 g/cm^2^). WQS mixture increased OP (OR = 1.20, 95% CI: 1.08, 1.32) and reduced lumbar BMD (β = −0.017). Stronger in females and ages 20–65.
Xu et al. [57]	2023	Sweden	Retrospective cohort study	61,504	Spatiotemporal environmental modeling	PFHxS, PFOS	ICD codes	**‘Ever-High’ PFAS:** Increased risk of MOF (HR = 1.11, 95% CI: 1.03, 1.19) and hip fractures (HR = 1.12, 95% CI: 1.00, 1.24). **‘Late-High’ PFAS:** Increased MOF (HR = 1.29, 95% CI: 1.16, 1.44), hip fractures (HR = 1.22, 95% CI: 1.01, 1.47), proximal humeral, and distal forearm fractures. Females ≥ 50 had higher MOF (HR = 1.18) and hip fracture (HR = 1.30) risks.
Xu et al. [58]	2022	United Kingdom	Cohort study	422,955	Spatiotemporal environmental modeling	PM2.5, PM10, PM2.5–10, NO2, NOx	ICD-10	**Air Pollution Score (per 10 Units):** Incident OP HR = 1.06 (95% CI: 1.03, 1.08). Individually: PM2.5 HR = 1.94 (95% CI: 1.52, 2.48), NO2 HR = 1.06, NOx HR = 1.03. PM10 and PM2.5–10 were null. **Genetic Synergy:** High PM2.5 + high genetic risk (PRS) increased OP risk (HR = 2.46, 95% CI: 2.2, −2.70; RERI = 0.17).
Yan et al. [59]	2023	China	Cross-sectional study	3385	Urinary biomonitoring	2,4,5-TCP, 2,4,6-TCP	DXA	**ln-2,4,5-TCP:** Marginal negative association strictly with lumbar spine BMD (β = −0.007, 95% CI: −0.013, −0.000, *p* = 0.04).
Yang et al. [60]	2025	United States	Cross-sectional study	3591	Urinary biomonitoring	11 PAE metabolites (MCNP, MCOP, MECPP, MBP, MCPP, MEP, MEHP, MHP, MiBP, MEOHP, and MBzP).	DXA	**MECPP (per log-unit):** Reduced total body (β = −0.022 g/cm^2^, *p* < 0.001), lumbar spine (β = −0.023 g/cm^2^, *p* = 0.001), and pelvic BMD (β = −0.026 g/cm^2^, *p* = 0.001). **MEHP & Other Phthalates:** MEHP positively correlated with total body (β = 0.016), lumbar spine (β = 0.030), and pelvic BMD (β = 0.034). Inverted U-shaped trends for MCNP, MECPP, MHP, and MEOHP (*p* < 0.05).
Yang et al. [61]	2023	United Kingdom	Cohort study	341,311	Spatiotemporal environmental modeling	PM2.5, PM10, PM2.5 absorbance, NO2, NOx	QUS and ICD-10 codes	**Cross-Sectional (per IQR):** Reduced eBMD (PM2.5: −0.0018, PM10: −0.0052, NO2: −0.0037, NOx: −0.0021 g/cm^2^, *p* < 0.001); elevated OP prevalence (PM2.5 OR = 1.05, PM10 OR = 1.08, NO2 OR = 1.07). **Incident OP (per IQR HR):** PM2.5 = 1.09 (95% CI: 1.06, 1.12), PM2.5 absorbance = 1.04, PM10 = 1.04, NO2 = 1.07, NOx = 1.06.
Yang et al. [62]	2025	China	Cross-sectional study	9870	Urinary biomonitoring	17 distinct metal elements (including Al, B, Cd, Co, Cr, Cu, Fe, Hg, Li, Mn, Mo, Ni, Pb, Sr, V, Zn, and Mg	QUS	**Urinary Metals (Q4 vs. Q1):** Ni (OR = 1.23, 95% CI: 1.01, 1.50) and Zn (OR = 1.56, 95% CI: 1.27, 1.90) increased hypertension-abnormal bone mass comorbidity risk. **BKMR Mixture:** Overall protective effect driven by vanadium and lithium, especially in adults ≥ 60 years.
Yu et al. [63]	2023	United Kingdom	Cohort study	430,120	Spatiotemporal environmental modeling	PM2.5, PM10, PM2.5–10, NO2, NOx	QUS and ICD-10 codes	**Individual Contaminants:** PM2.5 (HR = 1.05, 95% CI: 1.03, 1.07), NO2 (HR = 1.03), and NOx (HR = 1.03) increased OP and fracture risk; decreased eBMD. **Air Pollution Score (APS):** Q5 vs Q1 increased OP (HR = 1.14, 95% CI: 1.07, 1.21) and fracture risk (HR = 1.08). Combined high APS + low GRS increased OP risk by 86.1% and fracture risk by 44.0%.
Zhang et al. [64]	2022	China	Cross-sectional study	1845	Spatiotemporal environmental modeling	PM2.5, PM10, SO2, NO2, CO, O3	DXA	**Per 10 µg/m^3^ Increase:** Femoral neck t-score decreased by 0.20 (95% CI: 0.04, 0.36) for PM2.5 and 0.31 (95% CI: 0.11, 0.51) for SO2; total hip t-score decreased for CO (β = −0.03, 95% CI: −0.05, −0.02). **Osteoporosis Risk (per 1 µg/m^3^ PM2.5):** OR = 1.05 (95% CI: 1.00, 1.11; Q4 vs. Q1 OR = 2.10, 95% CI: 1.13, 3.91), stronger in males (OR = 1.29). PM10, NO2, and O3 were null.
Zhang et al. [65]	2024	China	Cross-sectional study	748	Spatiotemporal environmental modeling	PM2.5, Sulfate, Nitrate, Ammonium, Organic Matter, Black Carbon	DXA	**Inorganic Components (per IQR):** Elevated OP risk for NO3- (OR = 1.65, 95% CI: 1.13, 2.30) and NH4+ (OR = 1.77, 95% CI: 1.26, 2.49). **BMD Reductions:** L1-L4 lumbar BMD decreased by 16.05 g/cm^2^ (SO42−), 28.19 g/cm^2^ (NO3−), and 28.08 g/cm^2^ (NH4+); femoral neck BMD decreased by 16.58 g/cm^2^ (NO3−) and 23.56 g/cm^2^ (NH4+). Persisted in age > 60 and postmenopausal women; PM2.5, BC, and OM were null.
Zhou et al. [66]	2025	United States	Cross-sectional study	3555	Urinary biomonitoring	VOCs	DXA and treatment history	**VOC Metabolites (per SD Increase in OP Risk):** 3,4-MHA (OR = 1.25, 95% CI: 1.02, 1.55), BPMA (OR = 1.18, 95% CI: 1.01, 1.38), and 3-HPMA (OR = 1.24, 95% CI: 1.03, 1.51). VOCM mixture OP OR = 1.46 (95% CI: 1.04, 2.05). **BMD Changes:** BPMA (β = −0.010 g/cm^2^) and CYMA (β = −0.016 g/cm^2^) reduced lumbar BMD; 2-HPMA increased hip BMD.
Zhou et al. [67]	2024	China	Cohort study	17,566	Spatiotemporal environmental modeling	PM2.5, Organic Matter, BC, nitrate, chloride, sulfate, ammonium, NO2, O3	ICD-10 codes	**PM2.5 Exposure:** Incident OP risk increased per IQR (HR = 1.69, 95% CI: 1.33, 2.15). **Chemical Fractions:** Organic Matter (OM) had largest effect size (HR = 1.97, 95% CI: 1.46, 2.65). Joint air pollutant mixture increased incidence (HR = 1.36, 95% CI: 1.14, 1.61), with OM carrying maximum weight.

## Data Availability

The data presented in this study are available on request from the corresponding authors.

## Supplementary Materials

The following supporting information can be downloaded at https://www.mdpi.com/article/10.3390/toxics14090790/s1. Supplementary Table S1: Full Search Strategy. Supplementary Table S2: PRISMA Checklist.

## References

[1] Han M., Ma A., Dong Z., Yin J., Shao B. (2023). Organochlorine Pesticides and Polycyclic Aromatic Hydrocarbons in Serum of Beijing Population: Exposure and Health Risk Assessment. Sci. Total Environ..

[2] Bora S.S., Gogoi R., Sharma M.R., Anshu, Borah M.P.P., Deka P., Bora J., Naorem R.S.S., Das J., Teli A.B. (2024). Microplastics and Human Health: Unveiling the Gut Microbiome Disruption and Chronic Disease Risks. Front. Cell. Infect. Microbiol..

[3] Jahedi F., Jaafarzadeh Haghighi Fard N. (2025). Micro- and Nanoplastic Toxicity in Humans: Exposure Pathways, Cellular Effects, and Mitigation Strategies. Toxicol. Rep..

[4] Moscato A., Longo M.V., Ferrante M., Fiore M. (2025). Trifluoroacetic Acid: A Narrative Review on Physico-Chemical Properties, Exposure Pathways, and Toxicological Concerns. Environments.

[5] Leonforte F., Nicosia V., Comite P., Morlino G., Mistretta A. (2026). Media Exposure and Its Association with Vaccine Attitudes, Intentions, and Hesitancy: Systematic Review. J. Med. Internet Res..

[6] Peluso J., Chehda A.M., Olivelli M.S., Ivanic F.M., Pérez Coll C.S., Gonzalez F., Valenzuela L., Rojas D., Cristos D., Butler M. (2023). Metals, Pesticides, and Emerging Contaminants on Water Bodies from Agricultural Areas and the Effects on a Native Amphibian. Environ. Res..

[7] Soares A., Bertolo R.A., Dias M.A., Fregona L.G.G., Romero J.M.D., Villa J.E.L., Montagner C.C. (2026). Per- and Polyfluoralkylated Substances (PFAS) and Other Emerging Contaminants in Groundwater from Central Urban Areas of São Paulo City, Brazil. Environ. Monit. Assess..

[8] Liu Q., Chen Z., Chen Y., Yang F., Yao W., Xie Y. (2021). Microplastics and Nanoplastics: Emerging Contaminants in Food. J. Agric. Food Chem..

[9] de Carvalho J.G.R., Augusto H.C., Ferraz R., Delerue-Matos C., Fernandes V.C. (2024). Micro(Nano)Plastic and Related Chemicals: Emerging Contaminants in Environment, Food and Health Impacts. Toxics.

[10] Singh K., Bjerregaard P., Man Chan H. (2014). Association between Environmental Contaminants and Health Outcomes in Indigenous Populations of the Circumpolar North. Int. J. Circumpolar Health.

[11] Yang M., Jalava P., Hakkarainen H., Roponen M., Leskinen A., Komppula M., Dong G.-P., Lao X.-Q., Wu Q.-Z., Xu S.-L. (2022). Fine and Ultrafine Airborne PM Influence Inflammation Response of Young Adults and Toxicological Responses in Vitro. Sci. Total Environ..

[12] Yu T., Zhang H., Xie J., Yu Y., Zhang D., Wu Y., Sun L., Tian N., Zhou Y., Zhang X. (2025). Deciphering the Toxicological Mechanisms of Per- and Polyfluoroalkyl Substances (PFAS) in Musculoskeletal Disorders via Integrated Network Toxicology and Molecular Docking. Environ. Int..

[13] Wang Y., Wang Y., Li R., Ni B., Chen R., Huang Y., Cheng R., Li P., Li H., Peng Y. (2024). Low-Grade Systemic Inflammation Links Heavy Metal Exposures to Mortality: A Multi-Metal Inflammatory Index Approach. Sci. Total Environ..

[14] Li L., Su Y., Huang J., Xu W. (2025). Polystyrene Microplastics Induces Senescence of Osteocytes by Activating the Cyclooxygenase-2 Signaling Pathway. Ecotoxicol. Environ. Saf..

[15] Posso M., Sala M. (2024). PROSPERO—Reasons for Its Existence and Why a Systematic Review and/or Meta-Analysis Should Be Registered. Cir. Esp. Engl. Ed..

[16] Ouzzani M., Hammady H., Fedorowicz Z., Elmagarmid A. (2016). Rayyan—A Web and Mobile App for Systematic Reviews. Syst. Rev..

[17] Barker T.H., Hasanoff S., Aromataris E., Stone J.C., Leonardi-Bee J., Sears K., Klugar M., Tufanaru C., Moola S., Liu X.-L. (2026). The Revised JBI Critical Appraisal Tool for the Assessment of Risk of Bias for Analytical Cross-Sectional Studies. JBI Evid. Synth..

[18] Stang A. (2010). Critical Evaluation of the Newcastle-Ottawa Scale for the Assessment of the Quality of Nonrandomized Studies in Meta-Analyses. Eur. J. Epidemiol..

[19] McGuinness L.A., Higgins J.P.T. (2021). Risk-of-Bias VISualization (Robvis): An R Package and Shiny Web App for Visualizing Risk-of-Bias Assessments. Res. Synth. Methods.

[20] Bai T., Li X., Zhang H., Yang W., Lv C., Du X., Xu S., Zhao A., Xi Y. (2024). The Association between Brominated Flame Retardants Exposure with Bone Mineral Density in US Adults: A Cross-Sectional Study of the National Health and Nutrition Examination Survey (NHANES) 2005–2014. Environ. Res..

[21] Banjabi A.A., Li A.J., Kumosani T.A., Yousef J.M., Kannan K. (2020). Serum Concentrations of Perfluoroalkyl Substances and Their Association with Osteoporosis in a Population in Jeddah, Saudi Arabia. Environ. Res..

[22] Beglarian E., Costello E., Walker D.I., Wang H., Alderete T.L., Chen Z., Valvi D., Baumert B.O., Rock S., Rubbo B. (2024). Exposure to Perfluoroalkyl Substances and Longitudinal Changes in Bone Mineral Density in Adolescents and Young Adults: A Multi-Cohort Study. Environ. Res..

[23] Chang E.-C., Liang F.-W., Huang J.-C., Chang H.-H., Huang S.-P., Chen S.-C., Wu C.-D., Huang Y.-C., Geng J.-H. (2025). Association of Fine Particulate Matter (PM2.5) and Wet-Bulb Globe Temperature (WBGT) with Osteoporosis Incidence: A Nationwide Prospective Cohort Study. Int. J. Med. Sci..

[24] Chang Q., Zhang M., Yu Q., Yu S., Tang Y., Pan G., Cheng Y., Qin J., Wang X., Xia Y. (2025). Association between Particulate Air Pollution, Physical Activity, and the Risk of Osteoporosis in the UK Biobank. Ecotoxicol. Environ. Saf..

[25] Cheng B., Pan C., Cai Q., Liu L., Cheng S., Yang X., Meng P., Wei W., He D., Liu H. (2024). Long-Term Ambient Air Pollution and the Risk of Musculoskeletal Diseases: A Prospective Cohort Study. J. Hazard. Mater..

[26] Colicino E., Pedretti N.F., Busgang S.A., Gennings C. (2020). Per- and Poly-Fluoroalkyl Substances and Bone Mineral Density: Results from the Bayesian Weighted Quantile Sum Regression. Environ. Epidemiol..

[27] Di D., Zhang R., Zhou H., Wei M., Cui Y., Zhang J., Yuan T., Liu Q., Zhou T., Wang Q. (2023). Joint Effects of Phenol, Chlorophenol Pesticide, Phthalate, and Polycyclic Aromatic Hydrocarbon on Bone Mineral Density: Comparison of Four Statistical Models. Environ. Sci. Pollut. Res..

[28] Du J., Cui H., Zhao Y., Xue H., Chen J. (2024). Exposure to Air Pollution Might Decrease Bone Mineral Density and Increase the Prevalence of Osteoporosis: A Mendelian Randomization Study. Osteoporos. Int..

[29] Fan S., Wu Y., Bloom M.S., Lv J., Chen L., Wang W., Li Z., Jiang Q., Bu L., Shi J. (2023). Associations of Per- and Polyfluoroalkyl Substances and Their Alternatives with Bone Mineral Density Levels and Osteoporosis Prevalence: A Community-Based Population Study in Guangzhou, Southern China. Sci. Total Environ..

[30] Fu X., Wang Y. (2025). Possible Implication of Exposure to VOCs with the Development of Osteoporosis in the North American Population According to NHANES. Ecotoxicol. Environ. Saf..

[31] Fukushi J., Tokunaga S., Nakashima Y., Motomura G., Mitoma C., Uchi H., Furue M., Iwamoto Y. (2016). Effects of Dioxin-Related Compounds on Bone Mineral Density in Patients Affected by the Yusho Incident. Chemosphere.

[32] Gao Y., Lu H., Zhou H., Tan J. (2024). Exploring the Impact of Polychlorinated Biphenyls on Comorbidity and Potential Mitigation Strategies. Front. Public Health.

[33] Gu L., Wang Z., Liu L., Luo J., Pan Y., Sun L., Wang H., Zhang W.-B. (2022). Association between Mixed Aldehydes and Bone Mineral Density Based on Four Statistical Models. Environ. Sci. Pollut. Res..

[34] Guo J., Wang S., Zhang Z., Luan M. (2024). Associations between Organophosphate Esters and Bone Mineral Density in Adults in the United States: 2011–2018 NHANES. Ecotoxicol. Environ. Saf..

[35] Guo J., Huang Y., Bian S., Zhao C., Jin Y., Yu D., Wu X., Zhang D., Cao W., Jing F. (2018). Associations of Urinary Polycyclic Aromatic Hydrocarbons with Bone Mass Density and Osteoporosis in U.S. Adults, NHANES 2005–2010. Environ. Pollut..

[36] Heo S., Kim H., Kim S., Choe S.-A., Byun G., Lee J.-T., Bell M.L. (2022). Associations between Long-Term Air Pollution Exposure and Risk of Osteoporosis-Related Fracture in a Nationwide Cohort Study in South Korea. Int. J. Environ. Res. Public Health.

[37] Hou G., Xiao Q., Ye Z., Liu S., Zhou S., Wang Y., Li W., Zhang Y., Lv H. (2024). Epidemiological Characteristics of Elderly Osteoporosis Fractures and Their Association with Air Pollutants: A Multi-Center Study in Hebei Province. Sci. Rep..

[38] Hu Y., Liu G., Rood J., Liang L., Bray G.A., De Jonge L., Coull B., Furtado J.D., Qi L., Grandjean P. (2019). Perfluoroalkyl Substances and Changes in Bone Mineral Density: A Prospective Analysis in the POUNDS-LOST Study. Environ. Res..

[39] Hwang J., Kim K., Ahn S., Lee D., Lee S.W., Kim H.-J., Kim K. (2025). Association of Long-Term Exposure to Ambient Air Pollution with Osteoporosis among Cancer Survivors: Results from the Korea National Health and Nutrition Examination Survey. Prev. Med..

[40] India-Aldana S., Saddiki H., Tamayo-Ortiz M., Margetaki K., Valvi D., Landero J., Petrick L., Mercado-García A., Baccarelli A., Téllez-Rojo M.M. (2025). Women’s Bone Health Trajectories from Pregnancy to Postpartum: Associations with Bone-Seeking Metal Exposure Mixtures during Pregnancy. Ecotoxicol. Environ. Saf..

[41] Ju M., Liu F., Deng T., Jia X., Xu W., Zhang F., Gong M., Li Y., Yin Y. (2025). Association between Air Pollution and Osteoporosis: A Mendelian Randomization Study. Medicine.

[42] Khalil N., Chen A., Lee M., Czerwinski S.A., Ebert J.R., DeWitt J.C., Kannan K. (2016). Association of Perfluoroalkyl Substances, Bone Mineral Density, and Osteoporosis in the U.S. Population in NHANES 2009–2010. Environ. Health Perspect..

[43] Kirk A.B., DeStefano A., Martin A., Kirk K.C., Martin C.F. (2023). A New Interpretation of Relative Importance on an Analysis of Per and Polyfluorinated Alkyl Substances (PFAS) Exposures on Bone Mineral Density. Int. J. Environ. Res. Public Health.

[44] Lin P.-I.D., Cardenas A., Rokoff L.B., Rifas-Shiman S.L., Zhang M., Botelho J., Calafat A.M., Gold D.R., Zota A.R., James-Todd T. (2024). Associations of PFAS Concentrations during Pregnancy and Midlife with Bone Health in Midlife: Cross-Sectional and Prospective Findings from Project Viva. Environ. Int..

[45] Liu J., Wang K. (2024). Disentangling the Relationship Between Urinary Metal Exposure and Osteoporosis Risk Across a Broad Population: A Comprehensive Supervised and Unsupervised Analysis. Toxics.

[46] Peng S., Zhang G., Wang D., He Z. (2025). The Impact of Heavy Metals on Osteoporosis in Postmenopausal Women. Front. Environ. Health.

[47] Puerto-Parejo L., Aliaga I., Canal-Macias M., Leal-Hernandez O., Roncero-Martín R., Rico-Martín S., Moran J. (2017). Evaluation of the Dietary Intake of Cadmium, Lead and Mercury and Its Relationship with Bone Health among Postmenopausal Women in Spain. Int. J. Environ. Res. Public Health.

[48] Qiao D., Pan J., Chen G., Xiang H., Tu R., Zhang X., Dong X., Wang Y., Luo Z., Tian H. (2020). Long-Term Exposure to Air Pollution Might Increase Prevalence of Osteoporosis in Chinese Rural Population. Environ. Res..

[49] Reeves K.W., Vieyra G., Grimes N.P., Meliker J., Jackson R.D., Wactawski-Wende J., Wallace R., Zoeller R.T., Bigelow C., Hankinson S.E. (2021). Urinary Phthalate Biomarkers and Bone Mineral Density in Postmenopausal Women. J. Clin. Endocrinol. Metab..

[50] Shen Z., Zhang F., Guan X., Liu Z., Zong Y., Zhang D., Wang R., Xue Q., Ma W., Zhuge R. (2024). Associations of Pyrethroid Exposure with Bone Mineral Density and Osteopenia in Adults. J. Bone Miner. Metab..

[51] Shin J., Kweon H.J., Kwon K.J., Han S.-H. (2021). Incidence of Osteoporosis and Ambient Air Pollution in South Korea: A Population-Based Retrospective Cohort Study. BMC Public Health.

[52] Sun X., Mao Y., Liu B., Gu K., Liu H., Du W., Li R., Zhang J. (2023). Mesenchymal Stem Cell-Derived Exosomes Enhance 3D-Printed Scaffold Functions and Promote Alveolar Bone Defect Repair by Enhancing Angiogenesis. J. Pers. Med..

[53] Sun H., Wan Y., Pan X., You W., Shen J., Lu J., Zheng G., Li X., Xing X., Zhang Y. (2024). Long-Term Air Pollution and Adverse Meteorological Factors Might Elevate the Osteoporosis Risk among Adult Chinese. Front. Public Health.

[54] Vitku J., Kolatorova L., Franekova L., Blahos J., Simkova M., Duskova M., Skodova T., Starka L. (2018). Endocrine Disruptors of the Bisphenol and Paraben Families and Bone Metabolism. Physiol. Res..

[55] Ward-Caviness C.K., Moyer J., Weaver A., Devlin R., Diaz-Sanchez D. (2022). Associations between PFAS Occurrence and Multimorbidity as Observed in an Electronic Health Record Cohort. Environ. Epidemiol..

[56] Wei W., Huo H., Song X., Wang D., Huang H., Jiang F. (2025). Individual and Mixed Effects of PFAS on Osteoporosis: Insights from Epidemiological and Bioinformatic Approaches. Toxicol. Lett..

[57] Xu L., Pinxten W., Vandereyt F., Falter M., Scherrenberg M., Kizilkilic S.E., Van Erum H., Dendale P., Kindermans H. (2023). Motivational Communication Skills to Improve Motivation and Adherence in Cardiovascular Disease Prevention: A Narrative Review. Clin. Cardiol..

[58] Xu C., Weng Z., Liu Q., Xu J., Liang J., Li W., Hu J., Huang T., Zhou Y., Gu A. (2022). Association of Air Pollutants and Osteoporosis Risk: The Modifying Effect of Genetic Predisposition. Environ. Int..

[59] Yan Z., Xiong X., Tao J., Wang S. (2023). Association of Bone Mineral Density with Trichlorophenol: A Population-Based Study. BMC Musculoskelet. Disord..

[60] Yang S., Zhang R., Zhang H., Lu Y. (2025). Ultrasound-Targeted Microbubble Destruction: A Prospective Strategy for Treating Cardiovascular Disease. Front. Cardiovasc. Med..

[61] Yang C., Yin L., Guo Y., Han T., Wang Y., Liu G., Maqbool F., Xu L., Zhao J. (2023). Insight into the Absorption and Migration of Polystyrene Nanoplastics in Eichhornia Crassipes and Related Photosynthetic Responses. Sci. Total Environ..

[62] Yang J., Feng Y. (2025). Urinary Phthalate Metabolites Associated with Bone Mineral Density in Adults: Data from the NHANES 2011–2018. Bone.

[63] Yu X.-H., Cao H.-W., Bo L., Lei S.-F., Deng F.-Y. (2023). Air Pollution, Genetic Factors and the Risk of Osteoporosis: A Prospective Study in the UK Biobank. Front. Public Health.

[64] Zhang F., Zhou F., Liu H., Zhang X., Zhu S., Zhang X., Zhao G., Li D., Zhu W. (2022). Long-Term Exposure to Air Pollution Might Decrease Bone Mineral Density T-Score and Increase the Prevalence of Osteoporosis in Hubei Province: Evidence from China Osteoporosis Prevalence Study. Osteoporos. Int..

[65] Zhang F., Zhu S., Di Y., Pan M., Xie W., Li X., Zhu W. (2024). Ambient PM2.5 Components Might Exacerbate Bone Loss among Middle-Aged and Elderly Women: Evidence from a Population-Based Cross-Sectional Study. Int. Arch. Occup. Environ. Health.

[66] Zhou H., Cui Z., Di D., Chen Z., Zhang X., Ling D., Wang Q. (2025). Connecting Volatile Organic Compounds Exposure to Osteoporosis Risk via Oxidative Stress Based on Adverse Outcome Pathway Methodology. J. Environ. Sci..

[67] Zhou H., Hong F., Wang L., Tang X., Guo B., Luo Y., Yu H., Mao D., Liu T., Feng Y. (2024). Air Pollution and Risk of 32 Health Conditions: Outcome-Wide Analyses in a Population-Based Prospective Cohort in Southwest China. BMC Med..

[68] Mousavibaygei S.R., Bisadi A., ZareSakhvidi F. (2023). Outdoor Air Pollution Exposure, Bone Mineral Density, Osteoporosis, and Osteoporotic Fractures: A Systematic Review and Meta-Analysis. Sci. Total Environ..

[69] Pang K.-L., Ekeuku S.O., Chin K.-Y. (2021). Particulate Air Pollution and Osteoporosis: A Systematic Review. Risk Manag. Healthc. Policy.

[70] Leonforte F., Mercogliano M., Nicosia V., Lo Bianco M., Leonardi R., Cavallaro A., Ruggieri M., Mistretta A. (2026). The Muscle Dysmorphia–Eating Disorders Link as a Public Health Concern: An Epidemiological Study of Symptom Dimensions and Social Isolation. Front. Public Health.

[71] Elonheimo H., Lange R., Tolonen H., Kolossa-Gehring M. (2021). Environmental Substances Associated with Osteoporosis–A Scoping Review. Int. J. Environ. Res. Public Health.

[72] Prada D., López G., Solleiro-Villavicencio H., Garcia-Cuellar C., Baccarelli A.A. (2020). Molecular and Cellular Mechanisms Linking Air Pollution and Bone Damage. Environ. Res..

[73] Chimienti M., Morlino G., Ndreca D., Stefanidhi K., Zerellari O., Shkreli R., Gjergji S., Leonforte F., Nicosia V., Mistretta A. (2026). Barriers to Accessing Healthcare for People with Disabilities:A Systematic Review. Front. Public Health.

[74] He S., Zhang K. (2025). Cadmium-Induced Bone Toxicity: Deciphering the Osteoclast–Osteoblast Crosstalk. Biology.

[75] Tang C., Lv X., Zou L., Rong Y., Zhang L., Xu M., Li S., Chen G. (2025). Cadmium Exposure and Osteoporosis: Epidemiological Evidence and Mechanisms. Toxicol. Sci..

[76] Turan S. (2021). Endocrine Disrupting Chemicals and Bone. Endocr. Disruptors.

[77] Park S.Y., Kong S.H., Kim K.J., Ahn S.H., Hong N., Ha J., Lee S., Choi H.S., Baek K.-H., Kim J.-E. (2024). Effects of Endocrine-Disrupting Chemicals on Bone Health. Endocrinol. Metab..

[78] Gogakos A.I., Polyzos S.A., Anastasilakis D.A., Anastasilakis A.D. (2026). Exploring the Relationship between Microplastic Exposure and Bone Health. Endocr. Connect..

[79] Zhao H., Mu S., Wang W., Li X. (2025). Potential Threats of Environmental Microplastics to the Skeletal System: Current Insights and Future Directions. Front. Endocrinol..

[80] Bai X., Wu F., Qin Y., Fu Q., Wang J., Pan Y. (2026). The Particle Size Effect: Cytotoxicity and Cellular Uptake of Polystyrene Nanoplastics in Human Keratinocytes. Toxics.

[81] Dzierżyński E., Blicharz-Grabias E., Komaniecka I., Panek R., Forma A., Gawlik P.J., Puźniak D., Flieger W., Choma A., Suśniak K. (2025). Post-Mortem Evidence of Microplastic Bioaccumulation in Human Organs: Insights from Advanced Imaging and Spectroscopic Analysis. Arch. Toxicol..

[82] Migliorati V., Simonetti F., Bertagnin L., Bodo E. (2026). Polypropylene Nanoplastics as PFAS Carriers: A Computational Study of the Adsorption Mechanism. Environ. Pollut..

[83] Wang S., Zhao X., Zhou R., Jin Y., Wang X., Ma X., Lu X. (2024). The Influence of Adult Urine Lead Exposure on Bone Mineral Densit: NHANES 2015-2018. Front. Endocrinol..

[84] Prabowo N.A., Soetrisno S., Nurwati I., Ardyanto T.D., Poncorini E., Nurudhin A., Dirgahayu P. (2025). Association between Air Pollution and Osteoporosis: A Systematic Review. BIO Web Conf..

